# ZmMS1 coordinates ROS homeostasis, lipid allocation, and male fertility for maize breeding applications

**DOI:** 10.1111/jipb.70303

**Published:** 2026-05-26

**Authors:** Quancan Hou, Xueli An, Zhenying Dong, Biao Ma, Suowei Wu, Ke Xie, Ziwen Li, Tingwei Yan, Yilin Jiang, Taotao Zhu, Bin Ma, Lina Zhao, Yan Long, Xiangyuan Wan

**Affiliations:** ^1^ Beijing Key Laboratory of Maize Bio‐breeding, School of Advanced Agricultural Sciences, Research Institute of Biology and Agriculture University of Science and Technology Beijing Beijing 100083 China; ^2^ Zhongzhi International Institute of Agricultural Biosciences Beijing 100192 China; ^3^ Beijing Engineering Laboratory of Main Crop Bio‐Tech Breeding, Beijing International Science and Technology Cooperation Base of Bio‐Tech Breeding Beijing Solidwill Sci‐Tech Co. Ltd. Beijing 100192 China

**Keywords:** hybrid breeding, LBD transcription factor, lipid allocation, maize, male‐sterile, pollen and anther, ROS homeostasis, ZmMS1

## Abstract

Identifying male‐sterile genes and developing biotechnology‐based male‐sterility systems are crucial for advancing hybrid maize breeding. However, this progress is hampered by the limited number of characterized key regulators and their incomplete mechanistic understanding in male sterility, as well as fertility instability of male‐sterile lines and a lack of efficient maintainer lines in maize. Here, we elucidate the multifaceted roles of ZmMS1, an LBD transcription repressor. ZmMS1 coordinates timely tapetal PCD by repressing ROS‐scavenging genes to regulate anther ROS homeostasis, while its DNA‐binding activity is redox‐sensitive, suggesting a potential redox‐dependent feedback mechanism. In addition, ZmMS1 balances lipid allocation between anther cuticle and pollen exine by directly repressing sporopollenin biosynthesis and indirectly promoting cutin/wax formation. Constitutive overexpression of *ZmMs1* induces dwarfism associated with GA and ABA homeostasis, and produces ~50% sterile and small pollen grains in maize and rice, offering a potential route for dwarf and male‐sterile breeding. Leveraging the findings that loss and precocious expression of *ZmMs1* cause recessive and dominant sterility, respectively, we develop a non‐transgenic multi‐control sterility system and a dominant genic male‐sterility system, both showing stable and complete sterility across diverse backgrounds without yield penalty, thereby providing flexible options for hybrid maize breeding. Our findings reveal that ZmMS1, as a redox‐sensitive transcription factor, regulates male fertility with previously unrevealed mechanisms, and provide practical tools for efficient hybrid maize breeding.

## Graphical abstract

The redox‐sensitive transcriptional repressor ZmMS1 coordinates reactive oxygen species homeostasis and lipid allocation for pollen exine and anther cuticle formation. Loss or precocious expression of *ZmMS1* causes male sterility. Constitutive overexpression induces dwarfism and semi‐sterility. These findings enable flexible maize male‐sterility systems for hybrid breeding

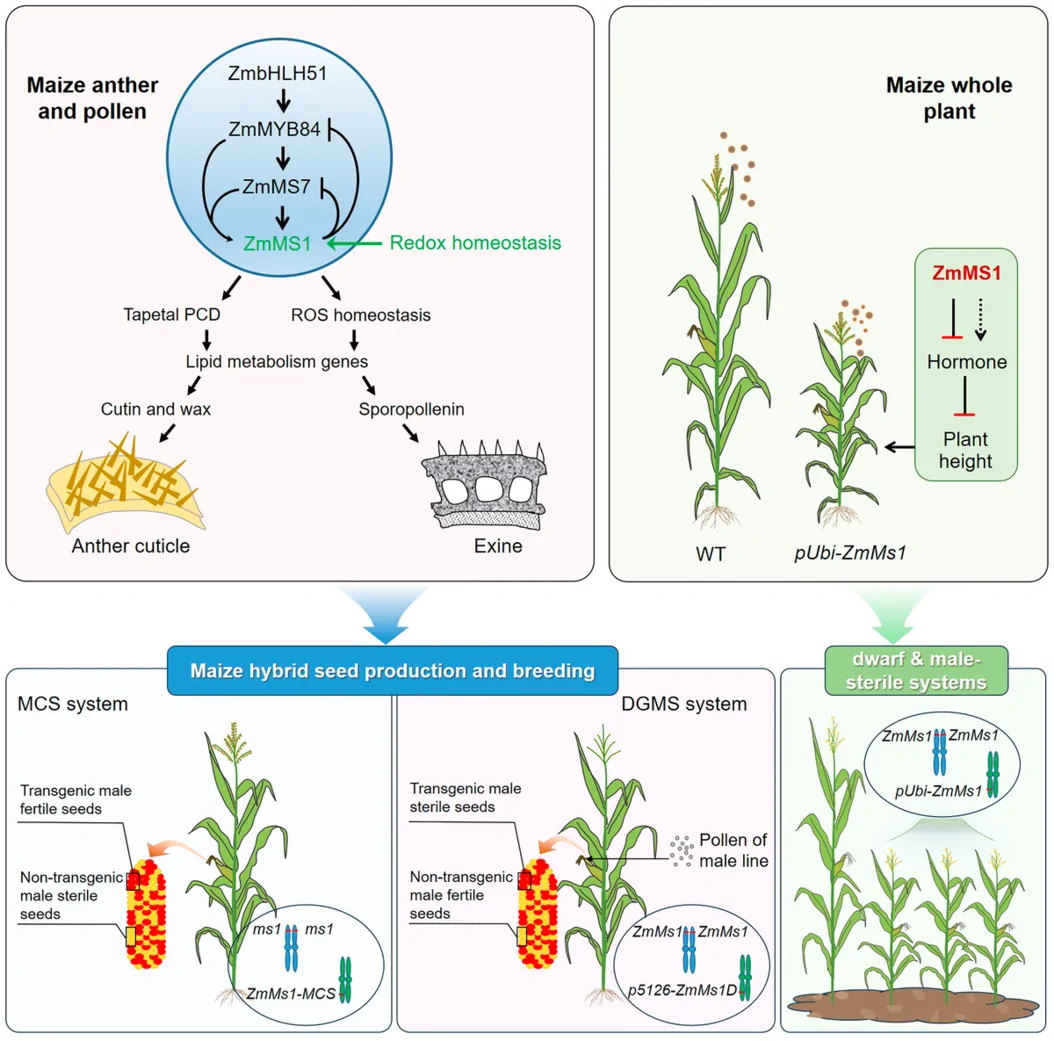

## INTRODUCTION

Maize is the crop with the largest planting area and yield in the world, and is vital for food, feed, and bioenergy. Heterosis is a phenomenon in which heterozygous hybrid progeny are superior to both homozygous parents, and the exploitation of heterosis through hybrid seed production is a cornerstone of modern maize breeding and greatly contributes to the global increase of maize grain yield (Wan et al., 2019; Liu et al., 2024; Paril et al., 2024). To produce pure hybrid seeds, female parent lines must be prevented from undergoing self‐pollination and be directionally crossed by male parent pollen because maize is a monoecious plant. Currently, manual or mechanical emasculation (detasseling), namely, the physical removal of the male floral structure, is used to prevent self‐pollination of female parent lines in many maize planting regions or countries such as China. However, this technique is not only time‐consuming, labor‐intensive, and expensive but also detrimental to plant growth, thus reducing the yield of hybrid seeds (Skibbe and Schnable, 2005; Zhang et al., 2018). Therefore, the use of male‐sterile lines as female parents is crucial to ensure efficient and cost‐effective cross‐pollination, and the identification and mechanism dissection of male‐sterile genes facilitate the development of biotechnology‐based male‐sterile (BMS) systems to promote hybrid seed production.

Anther and pollen development is a highly orchestrated process involving precise transcriptional programs, metabolic coordination, and timely cellular differentiation and degradation (Nelms and Walbot, 2019, 2022; Marchant and Walbot, 2022; Hou et al., 2025; Liu et al., 2026). The tapetum, the innermost somatic layer of four anther wall layers, plays a central secretory role in pollen wall formation and anther cuticle development by providing enzymes, nutrients, lipidic precursors, and regulatory siRNAs (Long et al., 2021; Yao et al., 2022). Its programmed cell death (PCD) at a specific anther developmental stage is critical for pollen maturity and anther dehiscence (Li et al., 2006; Hou et al., 2023). Reactive oxygen species (ROS) are key signals triggering tapetal PCD (Xie et al., 2014; Zheng et al., 2019; Zhu et al., 2020; Zhang et al., 2024). Consequently, the tight spatiotemporal control of ROS homeostasis by balancing ROS production and scavenging is essential for plant male fertility (Hu et al., 2011; Jacobowitz et al., 2019; Fang et al., 2023; Wu et al., 2023). Beyond their signaling roles, ROS can directly modulate protein function by oxidizing redox‐sensitive residues, such as cysteines, thereby influencing the activities of transcription factors and enzymes, a regulatory layer observed in various plant developmental contexts (Shaikhali et al., 2012; Viola et al., 2013; Tian et al., 2018). However, it remains unclear whether the ROS burst to trigger tapetal PCD directly regulates protein function to ensure proper anther and pollen development.

The lipidic materials used to synthesize sporopollenin for pollen exine and cutin/wax for anther epidermal cuticle are largely derived from the tapetum (Li et al., 2019a; Wan et al., 2020; Jiang et al., 2021b; Qiao et al., 2023; An et al., 2025). These biosynthetic pathways of sporopollenin, cutin, and wax share common precursors and enzymes, implying that a tightly regulated metabolic partitioning may exist to allocate limited lipid resources appropriately by bifurcating into two pathways for pollen exine and anther cuticle development, respectively (Wan et al., 2020). Any disruption of this balance often leads to defective pollen or anther integrity, thereby resulting in plant male sterility (Liu et al., 2022; An et al., 2024). Although several transcription factors regulating parts of these lipid biosynthetic pathways have been identified (Ranjan et al., 2017; Yang et al., 2019; An et al., 2020; Jiang et al., 2021a), the master regulators that coordinate the lipid allocation between pollen exine and anther cuticle remain elusive.

Currently, male‐sterile systems used in maize hybrid seed production include cytoplasmic male sterility (CMS) and genic male sterility (GMS). However, the S‐CMS (derived from the United States Department of Agriculture (USDA) germplasm) is susceptible to environmental instability and the T‐CMS (first identified in Texas, USA) often suffers from disease susceptibility (Levings, 1993; Weider et al., 2009). In addition, GMS genes lack maintainer lines via the traditional breeding strategies (Wan et al., 2019). Therefore, so far, few CMS and GMS genes have been commercially used for maize hybrid seed production in conventional maize breeding. Fortunately, the rapid development of gene cloning, gene editing, and genetic transformation techniques in maize has provided new possibilities for creating genetically engineered male sterility. For instance, several maize GMS genes have been used in developing BMS systems, such as *ZmMs26* and *ZmMs45* in the seed production technology (SPT) system (Wu et al., 2016; Qi et al., 2020), *ZmMs7*, *ZmMs30*, and *ZmMs33* in the multi‐control sterility (MCS) system (Zhang et al., 2018; An et al., 2019; Zhu et al., 2019), and *ZmMs44* and *ZmMs7* with *p5126* promoter in the dominant genic male sterility (DGMS) system, which can enhance maize grain yield (Fox et al., 2017; An et al., 2020; Wan et al., 2021). Nevertheless, some of these BMS systems have risks of transgenic pollen transmission or cannot generate pure non‐transgenic male‐sterile seeds, which prevents their entry into markets in strict GMO regulation regions or countries. Moreover, these BMS systems are typically designed solely for conferring sterility and not coupled with other beneficial agronomic traits.

Crop dwarfing breeding, exemplified by the green revolution genes like *Rht* in wheat (Würschum et al., 2017; Song et al., 2023) and *sd1* in rice (Spielmeyer et al., 2002; Hedden, 2003), has dramatically improved yield by enhancing lodging resistance and harvest index. Dwarf & male‐sterile (DMS) systems have been continuously refined and applied with considerable success in wheat and rice breeding (Zhai and Liu, 2009; Ansari et al., 2017; Xia et al., 2017; Li et al., 2019b; Zheng et al., 2025). Therefore, identifying maize genes that can concurrently induce male sterility and reduce plant height enables the development of a DMS system, which is valuable, yet underexplored in maize breeding.

Most LBD family proteins are primarily known to regulate plant vegetative development, with only a few implicated in reproductive processes (Majer and Hochholdinger, 2011; Zhang et al., 2020). ZmMS1, an LBD transcription factor, has recently been characterized as a key repressor of a transcriptional activation cascade, ZmbHLH51–ZmMYB84–ZmMS7, essential for maize male fertility (Hou et al., 2023; Zhou and Dobritsa, 2023). It displays highly specific temporal expression in maize anther and acts as a molecular brake to efficiently repress crucial regulators of tapetal PCD and sporopollenin biosynthesis, thereby revealing a mechanism for precise exine formation and marking a milestone in constructing a comprehensive molecular regulatory network map for the entire process of plant male development (Zhou and Dobritsa, 2023). However, the detailed functional mechanisms of *ZmMs1* underlying ROS homeostasis in anther and lipid allocation synthesized in tapetal cells, and its potential pleiotropic effects on vegetative and reproductive development when artificially manipulating its ectopic or precocious expression, remain to be elucidated.

This study investigates novel functions of ZmMS1 in regulating anther ROS homeostasis and balancing the lipid allocation between anther cuticle and pollen exine, and also discovers its unexpected effects on plant architecture and male fertility when it is constitutively overexpressed in maize whole plants. Based on these fundamental research findings, we develop *ZmMs1*‐driven MCS and DGMS systems, and evaluate their efficacy and stability, and also propose a DMS system for hybrid maize breeding. Thus, our work provides novel insights into anther/pollen development and plant architecture regulation, and delivers practical tools for modern maize breeding.

## RESULTS

### ZmMS1 controls ROS homeostasis by repressing ROS‐related genes in maize anther

Neither loss of *ZmMs1* function nor precocious expression of *ZmMs1* driven by an anther‐specific promoter (*p5126*, active during anther developmental stages S6 to S9–10) causes any abnormal phenotype in vegetative growth, but both result in complete male sterility in maize. In both materials, neither exserted anthers nor mature pollen grains were observed (Figure 1A). Transmission electron microscopy (TEM) analysis of anther wall layers indicated that tapetum degeneration was delayed in the *ms1‐alb* mutant, but occurred earlier in *p5126‐ZmMs1* (#1) compared to the wild type (WT) (Figure S1). Consistent with this change trend in the timing of tapetal PCD, tapetal cell binucleation occurred later in *ms1‐alb,* but earlier in *p5126‐ZmMs1* (#1) than in WT (Figure 1B), underscoring the critical role of precise *ZmMs1* expression in anther development. ROS level is an important factor triggering tapetal PCD (Xie et al., 2014). We measured total ROS levels using 2’,7’‐Dichlorodi‐hydrofluorescein diacetate (H_2_DCFDA) staining. In WT anthers, ROS levels peaked at S9 and S9–10 (Figures 1C, D, S2 and S3), aligning with the ROS requirement for tapetal PCD, which typically initiates at S9–10 (Hou et al., 2023). However, ROS‐level peaks shifted to S10 and S11 in *ms1‐alb* anthers and to S7 and S8 in *p5126‐ZmMs1* (#1) anthers (Figures 1C, D, S2 and S3), corresponding to the delayed and premature tapetal PCD observed in *ms1‐alb* and *p5126‐ZmMs1* (#1) anthers, respectively.

**Figure 1:**
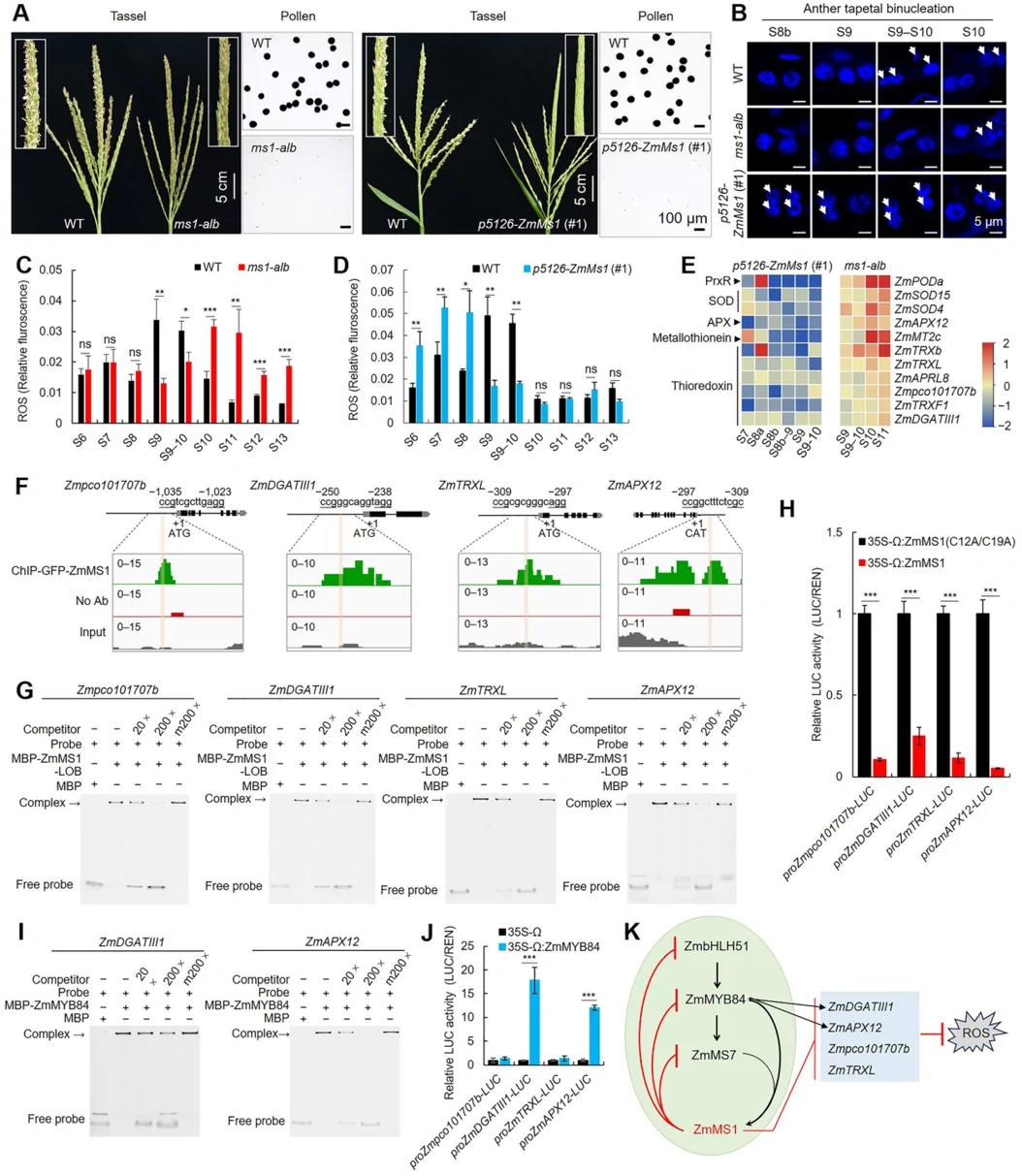
***ZmMs1*** **controls ROS levels by repressing ROS‐related genes in maize anther** **(A)** Tassels and pollen grains (stained by I_2_‐KI) of the *ms1‐alb* mutant, the *p5126‐ZmMs1* (#1) transgenic maize line, and their corresponding wild types (WT) with B73 for *ms1‐alb* and Hi II for *p5126‐ZmMs1* (#1). **(B)** DAPI staining of anther sections from anther developmental stages S8b to S10 in WT, *ms1‐alb*, and *p5126‐ZmMs1* (#1), showing tapetal cell binucleation. White arrows indicate binucleated tapetal cells. **(C**, **D)** ROS levels measured by 2’,7’‐dichlorodihydrofluorescein diacetate (H_2_DCFDA) staining in *ms1‐alb* and B73 anthers **(C)**, and *p5126‐ZmMs1* (#1) and Hi II anthers **(D)** from S6 to S13. **(E)** Heatmap showing expression changes of ROS‐related genes in *ms1‐alb* anthers (S9 to S11) and *p5126‐ZmMs1* (#1) anthers (S7 to S9–10) compared to their WT anthers by transcriptome analysis. Log_2_ expression changes relative to WT are shown and the color bar indicates the Log_2_ fold change scale. **(F)** ChIP‐seq peaks showing the enrichment of ZmMS1 in the promoters of four ROS‐related genes, *Zmpco101707b*, *ZmDGATIII1*, *ZmTRXL*, and *ZmAPX12*. **(G)** EMSA analysis showing ZmMS1‐LOB binding to the promoters of *Zmpco101707b*, *ZmDGATIII1*, *ZmTRXL*, and *ZmAPX12*. **(H)** ZmMS1 represses the promoter activities of *Zmpco101707b*, *ZmDGATIII1*, *ZmTRXL*, and *ZmAPX12*. **(I)** EMSA analysis showing ZmMYB84 binding to the promoters of *ZmDGATIII1* and *ZmAPX12*. **(J)** ZmMYB84 activates the promoter activities of *ZmDGATIII1* and *ZmAPX12*. **(K)** Schematic diagram of the ZmMS1‐driven feedback repression loop controlling ROS homeostasis by regulating ROS‐related genes. Data in **(C**, **D**, **H**, **J)** represent means ± *SD* (*n* = 3) and asterisks indicate significant differences (*P < 0.05, **P < 0.01, and ***P < 0.001). A two‐tailed Student's *t*‐test was used to determine *P* values. ns, not significant.

RNA‐seq data analysis of *ms1‐alb* and *p5126‐ZmMs1* (#1) anthers revealed differentially expressed genes (DEGs) between *ms1‐alb*, *p5126‐ZmMs1* (#1) and their respective WTs (Datasets S1–S6). Few DEGs were identified between *ms1‐alb* and WT before S9, whereas a large number of DEGs were detected between *p5126‐ZmMs1* (#1) and WT as early as S5 (Figure S4). This is supported by the expression patterns of *ZmMs1* in these genetic materials. Notably, the number of DEGs between *p5126‐ZmMs1* (#1) and WT decreased at S8b. This decrease is likely due to the short time interval between S8a (meiosis I) and S8b (meiosis II), as well as the similar cellular activities at these two stages. Furthermore, this observation confirms that *ZmMs1* is not involved in regulating meiosis, consistent with the cytological analysis from our previous study (Hou et al., 2023). Gene Ontology analysis of upregulated DEGs in *ms1‐alb* from S8b–9 to S10 showed significant enrichment of the term “response to hydrogen peroxide”, suggesting that *ZmMs1* may be involved in ROS‐related metabolic pathways. Among the DEGs, many ROS‐scavenging genes were upregulated in *ms1‐alb* during later stages (S9 to S11), but downregulated in *p5126‐ZmMs1* (#1) from S7 to S9–10 (Figures 1E, S4; Dataset S7), suggesting that ZmMS1 represses their transcription. These ROS‐scavenging genes encode different types of proteins, including peroxiredoxins, superoxide dismutases, ascorbate peroxidases, metallothioneins, and thioredoxins (Figure 1E), indicating a broad regulatory role of ZmMS1 in ROS metabolism. ChIP‐seq analysis showed that ZmMS1 enriched the promoter fragments of *Zmpco101707b*, *ZmDGATIII1*, *ZmTRXL*, and *ZmAPX12* (Figure 1F), all of which contain the ZmMS1‐binding motif. EMSA (electrophoretic mobility shift assay) and TDLR (transient dual‐luciferase reporter) assays confirmed that ZmMS1 binds to these promoters and significantly represses their activities (Figure 1G, H). Additionally, ZmMYB84 bound to and activated the promoters of *ZmDGATIII1* and *ZmAPX12* (Figure 1I, J). Together, the feedback suppression loop of ZmbHLH51–ZmMYB84–ZmMS7–ZmMS1 (Hou et al., 2023) fine‐tunes the expression of ROS‐scavenging genes, modulates anther ROS homeostasis, and ensures the correct timing of tapetal PCD, which is essential for male fertility in maize (Figure 1K).

### Homodimerization of ZmMS1 and redox status are crucial for DNA‐binding capacity

Proteins of LBD family contain a conserved N‐terminal LOB domain and a variable C‐terminal domain (Majer and Hochholdinger, 2011). The N‐terminal LOB domain of ZmMS1 recognizes a DNA sequence of CTCCGNNNNNNNCGGAG, with the CX_2_CX_6_CX_3_C motif being critical for DNA binding. In this motif, four cysteine residues (Cys9, 12, 19, and 23) form a zinc finger essential for zinc ion coordination (Figure 2A; Hou et al., 2023). Given the palindromic nature of its recognition sequence, we hypothesized that ZmMS1 functions as a homodimer. Yeast two‐hybrid assay showed that co‐expression of GAL4–BD–ZmMS1–LOB and GAL4–AD–ZmMS1–LOB fusion proteins can occur on quadruple dropout media (Figure 2B), indicating ZmMS1 dimerization via the LOB domain. We further performed a luciferase complementation assay (LCA) to validate the LOB domain dimerization *in planta*. The transfected tobacco epidermis leaf cells expressing *ZmMs1–LOB–nLUC* and *cLUC–ZmMs1–LOB* displayed luciferase activity (Figure 2C). In addition, the predicted three‐dimensional structure of the ZmMS1–LOB dimer aligned closely with the crystal structure of wheat Ramosa2 LOB domain dimer (pdb: 5ly0) (Figure S5A). These results confirm that ZmMS1 forms homodimers via its LOB domain. Molecular docking using HADDOCK generated a structural model of the ZmMS1–LOB dimer, which can bind to double‐strand DNA (Figure 2D). According to the modeled structure, the two zinc fingers penetrate the major grooves of the double‐stranded DNA. The residue Gln‐15 and positively charged residues Arg‐16, Arg‐17, and Lys‐18 are key residues of the interaction interface and form hydrogen bonds with the binding core bases (CTCCG) on both strands (Figure 2D).

**Figure 2:**
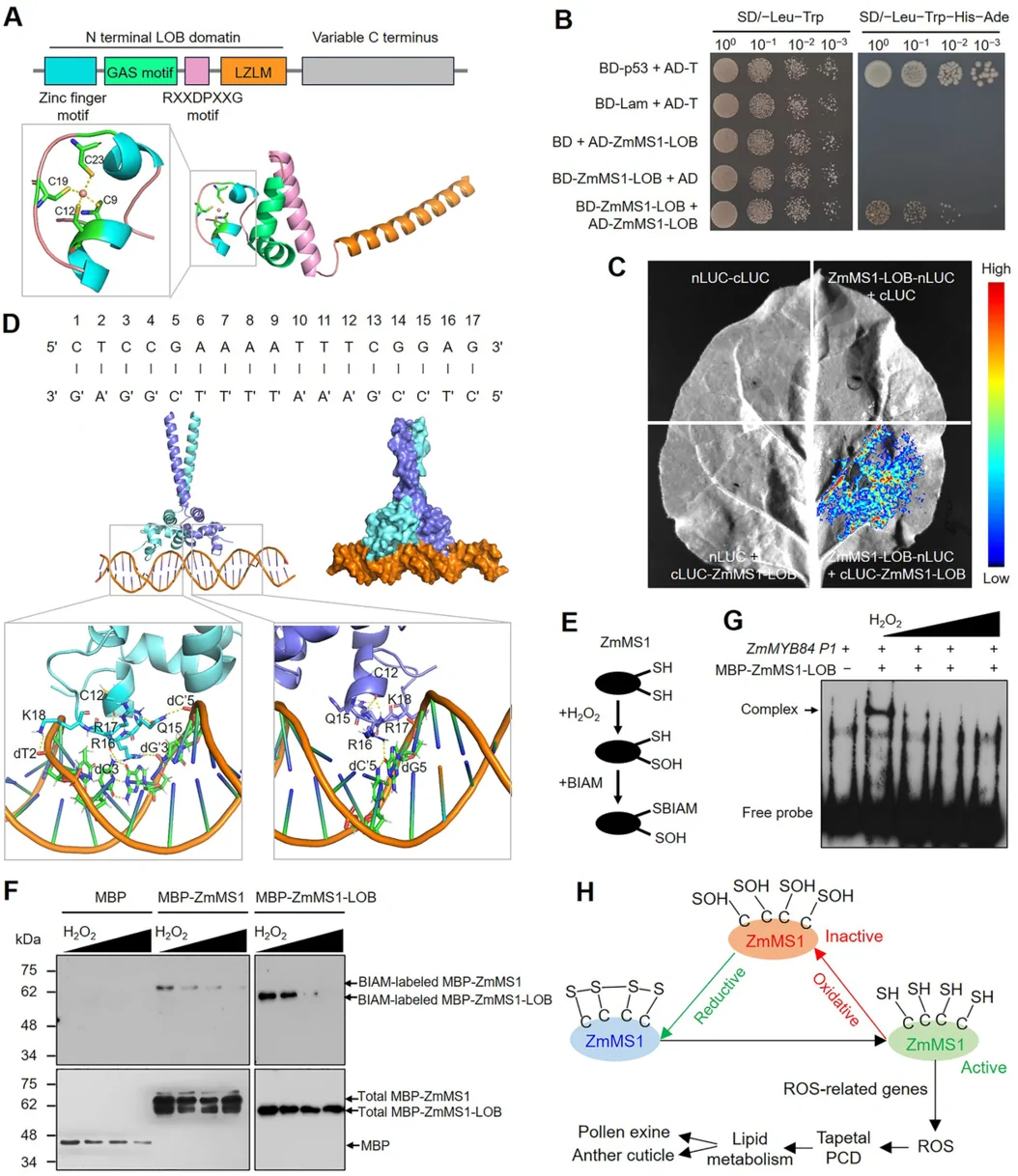
Homodimerization of ZmMS1 and redox status are crucial for the DNA‐binding capacity of ZmMS1 **(A)** Conserved protein domains of ZmMS1 and predicted three‐dimensional structure of its LOB domain. **(B**, **C)** Homodimerization of the LOB domain of ZmMS1 determined by yeast two‐hybrid **(B)** and luciferase complementation **(C)** assays. BD‐P53 + AD‐T and BD‐Lam + AD‐T in **(B)** serve as positive and negative controls, respectively. **(D)** Modeled ZmMS1–LOB–DNA complex structure. In the upper panel, the DNA‐binding motif of ZmMS1 (5’‐CTCCGAAAATTTCGGAG‐3’) is shown. In the middle panel, the ZmMS1–LOB homodimer and its binding DNA complex structures are modeled and shown for surface and cartoon, respectively. Two ZmMS1–LOB monomers are colored in purple and cyan, respectively. DNA is colored in orange. The bottom panel shows enlarged views of the two zinc fingers in contact with the major groove of the binding DNA. Hydrogen bonds between the highly conserved amino acids and the DNA‐binding motif are shown. **(E)** Workflow for quantifying redox modification of ZmMS1 using BIAM labeling. **(F)** Oxidative modification of ZmMS1 and its LOB domain (ZmMS1–LOB) by the BIAM‐labeling assay. The labeled proteins were detected by gel blot analysis with HRP‐conjugated streptavidin and MBP (maltose‐binding protein) antibodies. **(G)** DNA‐binding analysis of the oxidized MBP–ZmMS1–LOB protein to the *ZmMYB84* promoter. **(H)** Schematic diagram of the interconversion of ZmMS1‐binding activities between reduced and oxidized states via disulfide bond formation among four cysteines. ZmMS1 is active when the four cysteines maintain their monothiols in a reduced state and can bind to promoters of its downstream target genes, including ROS‐related genes, to ensure anther and pollen development.

Four variant versions of purified maltose‐binding protein (MBP)‐tagged ZmMS1–LOB protein lost DNA‐binding capacity when any of the four cysteines (Cys9, 12, 19, and 23) in the LOB domain was mutated to alanine (Figure S5B, C), indicating that these four cysteines are critical for DNA binding. Mass spectrometric analysis of disulfide bonds in tryptic fragments from purified ZmMS1 protein revealed that these four cysteines can form inter‐ and intra‐molecular disulfide bonds, and these disulfide bonds were eliminated under reduction conditions upon DTT treatment (Figure S5D). The biotin‐conjugated iodoacetamide (BIAM)‐labeling assay showed that MBP‐tagged ZmMS1 and ZmMS1–LOB could be labeled by BIAM and the labeling levels were decreased by H_2_O_2_ treatment in a dose‐dependent manner (Figure 2E, F). Moreover, H_2_O_2_ treatment significantly impaired the DNA‐binding capacity of ZmMS1–LOB (Figure 2G). These results suggest that oxidation of cysteine residues disrupts the LOB domain conformation, thereby disturbing its DNA‐binding capacity. Therefore, the redox status of these cysteine residues is crucial for the DNA‐binding activity of ZmMS1 to regulate the expression of ROS‐ and lipid metabolism‐related genes, which suggests a potential redox‐dependent feedback mechanism to ensure pollen exine formation and anther cuticle development required for male fertility in maize (Figure 2H).

### The ZmMS1‐driven feedback repression loop balances anther cuticle and pollen exine formation

The biosynthesis of sporopollenin, cutin, and wax in the tapetum shares several conserved pathways, and a reciprocal relationship exists between sporopollenin biosynthesis for pollen exine and cutin/wax biosynthesis for anther cuticle (Wan et al., 2020). Besides the dramatically thicker exine, *ms1‐alb* anthers showed a smooth outer surface (Figure 3A), indicating defective cuticle formation. Consistent with this phenotype, the cutin and wax contents were significantly lower in *ms1‐alb* compared to WT anthers (Figure 3B) and a wax biosynthesis‐related gene *ZmKCS‐4* was significantly downregulated at S10, the critical stage for anther cuticle formation (Figure 3C). Conversely, *myb84* mutant anthers displayed a thinner exine, but a denser epidermal cuticle layer at S10 and thereafter (Figure 3A–D). *ZmPKSB*, a direct target gene of ZmMYB84, forms a regulatory module with ZmMYB84 to modulate cuticle–exine trade‐off in maize anthers (Liu et al., 2022). Similar to *myb84*, the *pksb* mutant showed a denser anther cuticle but thinner exine compared to WT (Figure 3A–D). Both cutin and wax contents were higher in *myb84* and *pksb* mutants than those in their WT lines (Figure S6A, B). These changes primarily resulted from the alterations of cutin and wax monomer amounts (Figure S6C). The misregulation of anther cuticle formation was confirmed by altered expression levels of cutin and wax biosynthesis‐related genes such as *ZmKCS‐4* and *ZmLTL1*. Both genes were downregulated in *ms1‐alb* anthers at S10, but upregulated in *myb84* and *pksb* anthers from S8b–9 to S9–10 (Figures 3C, S6D). In contrast, sporopollenin biosynthetic genes such as *ZmAPV1*, *ZmACOS5‐2*, *ZmPKSA‐1*, *ZmPKSA‐2*, and *ZmMs25* were upregulated in *ms1‐alb* anthers at S10 and S11, but downregulated in *myb84* and *pksb* anthers from S9 to S11 (Figures 3E, S6E). Among these genes, ChIP‐seq analysis showed promoters of *ZmACOS5‐2*‐ and *ZmPKSA‐1*‐enriched ZmMS1 (Figure S6F), indicating that these two genes might be the direct targets of ZmMS1. Furthermore, *p5126‐ZmMs1* (#1) anthers showed a thinner pollen exine and a smooth epidermal cuticle surface compared to WT (Figure 3F). Gene Ontology (GO) functional annotation of differential expression genes (DEGs) between *ms1‐alb* and *p5126‐ZmMs1* (#1) vs their WT lines by using RNA‐Seq data confirmed that ZmMS1 was involved in pollen exine formation and sporopollenin biosynthesis (Figures S7, S8). 28 representative lipid metabolic genes were upregulated in *ms1‐alb* anthers from S9 to S12, but downregulated in *p5126‐ZmMs1* (#1) anthers from S8a to S9 (Figure 3H). These findings indicate that ZmMS1, as a transcriptional repressor, balances lipidic component biosynthesis to ensure proper pollen exine and anther cuticle formation. Precocious expression of *ZmMs1* (*p5126‐ZmMs1*) impairs lipid metabolism, leads to lipid deficiency, and ultimately results in thinner exine and a smooth anther cuticle, whereas loss of *ZmMs1* function releases ZmMS1 repression and causes excess lipid accumulation and biased allocation to exine formation, yielding thicker exine but also a smooth anther cuticle.

**Figure 3:**
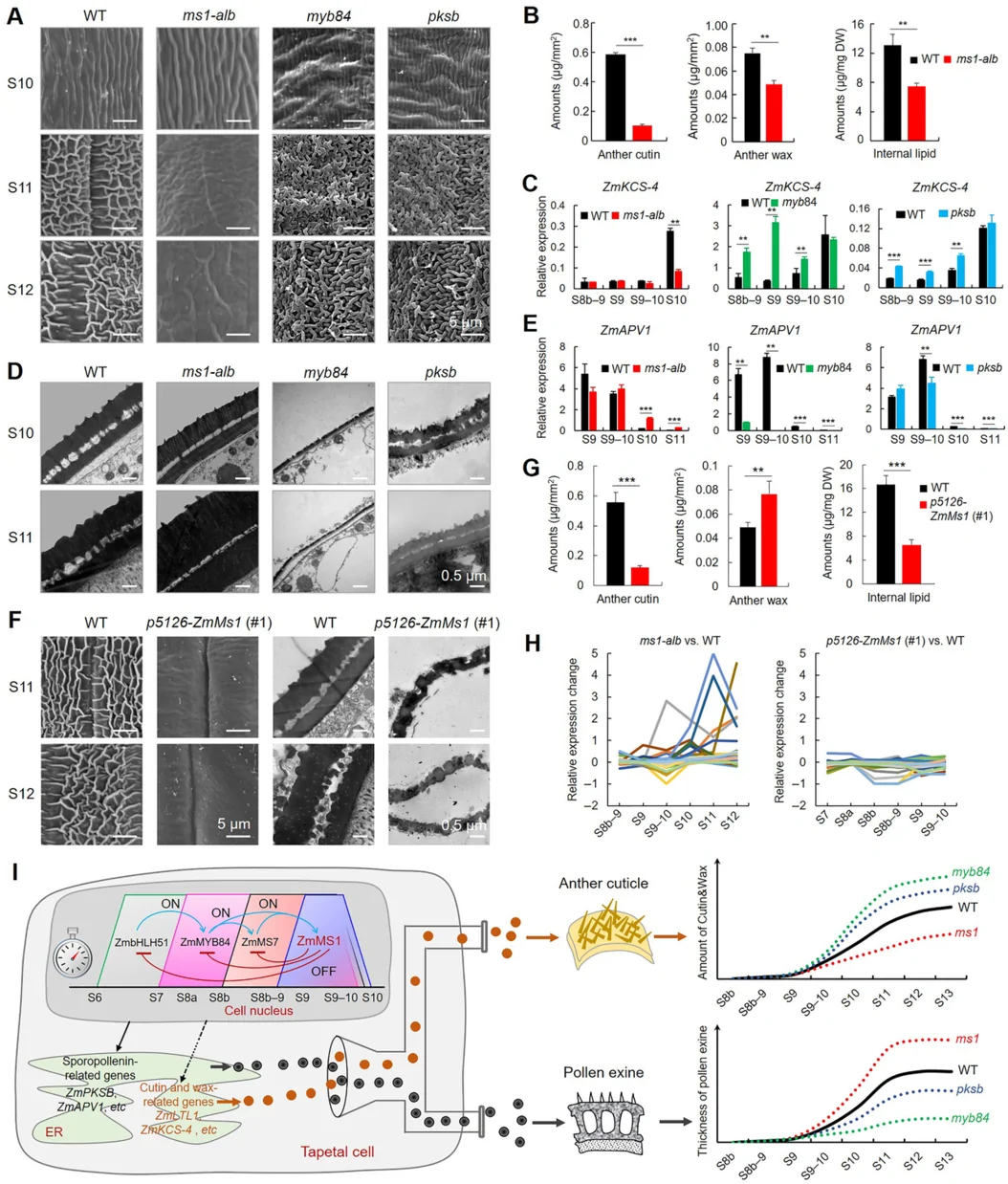
The ZmMS1‐driven feedback repression network balances anther cuticle and pollen exine formation **(A)** Comparison of anther cuticle of *ms1‐alb*, *myb84*, and *pksb* mutants and WT (B73, homozygous *ZmMs1* siblings) based on SEM observation from S10 to S12. **(B)** Anther internal lipid, epidermal cutin, and wax contents in *ms1‐alb* and WT anthers. **(C)** Expression changes of a wax‐related gene (*ZmKCS‐4*) in *ms1‐alb*, *myb84*, and *pksb* mutants and WT anthers from S8b–9 to S10 by RT‐qPCR analysis. **(D)** Comparison of pollen exine of *ms1‐alb*, *myb84*, and *pksb* mutants and WT based on TEM observation at S10 and S11. **(E)** Expression changes of a sporopollenin‐related gene (*ZmAPV1*) in *ms1‐alb*, *myb84*, and *pksb* and WT anthers from S9 to S11 by RT‐qPCR analysis. **(F)** Comparison of anther cuticle and pollen exine of *p5126‐ZmMs1* (#1) and WT (Hi II) at S11 and S12 based on SEM and TEM observation, respectively. **(G)** Anther internal lipid, epidermal cutin, and wax contents in *p5126‐ZmMs1* (#1) and WT anthers. **(H)** Expression changes of 28 genes related to lipid metabolism in *ms1‐alb* and *p5126‐ZmMs1* (#1) anther transcriptomes. **(I)** A working model of the ZmMS1‐driven feedback repression network balancing anther cuticle and pollen exine formation by directly regulating sporopollenin‐related genes and indirectly affecting anther cutin‐ and wax‐related genes. Data in **(B**, **C**, **E**, **G)** represent means ± *SD* (*n* = 3) and asterisks indicate significant differences (**P < 0.01 and ***P < 0.001). A two‐tailed Student's *t*‐test was used to determine *P* values.

Collectively, we propose that the ZmMS1‐driven feedback loop directly regulates sporopollenin‐related genes and indirectly influences cutin‐ and wax‐related genes, serving as a balancer for normal pollen exine and anther cuticle development in maize (Figure 3I). Loss of *ZmMs1* function releases the ZmMS1‐mediated repression of the ZmbHLH51–ZmMYB84–ZmMS7 cascade, leading to prolonged and elevated expression of sporopollenin‐related genes. This ultimately promotes the biosynthesis of excess sporopollenin precursors in the tapetum, resulting in thicker exine in the *ms1‐alb* mutant. Conversely, the expression of anther cutin‐ and wax‐related genes is indirectly activated by ZmMS1 through its repressed target genes such as *ZmMYB84* and *ZmPKSB*, particularly at stage S10, explaining the reduced cuticle lipids and the smooth outer surface of *ms1‐alb* anthers. Mutants of *ZmMYB84* and *ZmPKSB* displayed opposite phenotypes to *ms1‐alb*, namely, thinner exine but a denser epidermal cuticle layer with higher cutin and wax contents, supporting this notion.

### Ectopic overexpression of *ZmMs1* leads to dwarfism in association with altered GA and ABA homeostasis

*ZmMs1* is specifically expressed in anthers. Its loss of function and precocious expression give rise to recessive and dominant male sterility, respectively (Hou et al., 2023; Figure 1A). Beyond this specific role, members of the LBD transcription factor family are known to participate in a wide range of plant developmental programs and environmental adaptations (Majer and Hochholdinger, 2011). To investigate the constitutive ectopic expression effects of *ZmMs1*, we generated overexpression lines using the maize *ubiquitin* promoter (*pUbi‐ZmMs1*) (Figure S9A). We obtained five independent transgenic lines, which displayed a consistent dwarf phenotype across different environments (Figures 4A, S9B, C). Plant height of these lines was reduced to approximately 50% of WT (Figures 4B, S9D), with corresponding decreases in internode length, ear height, and tassel length (Figures 4C, S9E–G), while the internode number remained unchanged (Figure S9H). Leaf angles of bottom and top leaves were significantly enlarged, while middle leaves (the 7th to 9th leaves) showed no significant change compared to WT (Figure S9I). Both cell number and cell length in the fifth internode epidermis of *pUbi‐ZmMs1* (#1) were significantly reduced compared to WT (Figure 4D–F). *ZmMs1* expression was barely detectable in WT stem, while its transcript abundance was significantly increased in *pUbi‐ZmMs1* (#1) stem (Figure 4G), indicating that the constitutive overexpression of *ZmMs1* causes dwarfism.

**Figure 4:**
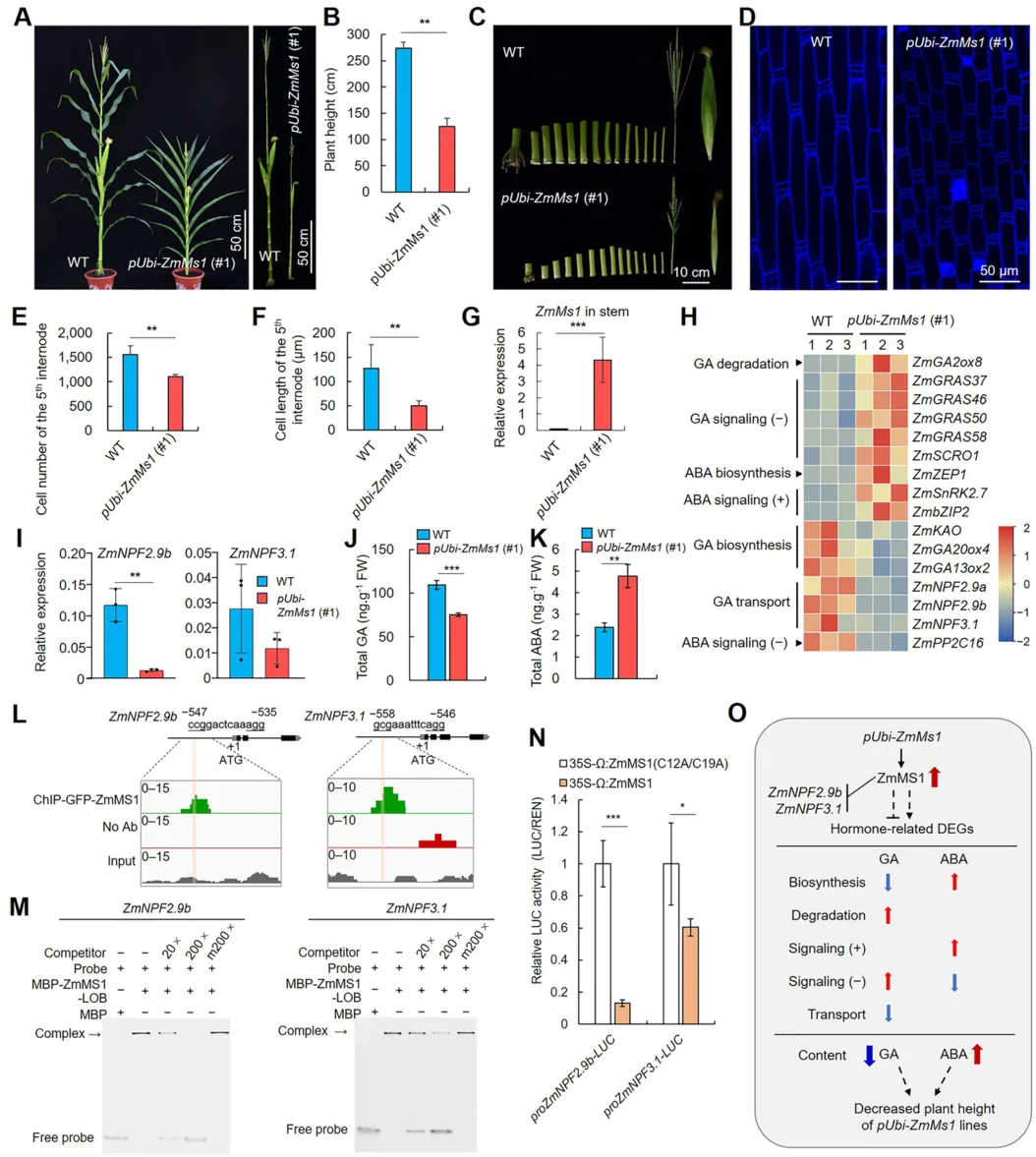
**Ectopic overexpression of** ***ZmMs1*** **reduces plant height by altering GA and ABA levels** **(A)** Whole‐plant phenotype of a *ZmMs1‐*overexpression line (*pUbi‐ZmMs1* (#1)) driven by the maize *ubiquitin* promoter. **(B)** Quantitative measurements of plant height of WT and the *pUbi‐ZmMs1* (#1) line at anthesis. **(C)** Comparison of tassels, ears, and aboveground internodes of WT and a *pUbi‐ZmMs1* (#1) line at anthesis. **(D)** Epidermal cell length of the fifth internode in WT and the *pUbi‐ZmMs1* (#1) line at the jointing stage. **(E**, **F)** Cell number **(E)** and cell length **(F)** of the fifth internode of WT and the *pUbi‐ZmMs1* (#1) line at the jointing stage. **(G)** *ZmMs1* expression in the fifth internode of WT and the *pUbi‐ZmMs1* (#1) line at the jointing stage by RT‐qPCR analysis. **(H)** Heatmap showing expression changes of GA‐ and ABA‐related genes based on the RNA‐seq data in WT and the *pUbi‐ZmMs1* (#1) line. Expression values are Log_2_‐transformed and the color bar indicates the scale. **(I)** Expression of two GA transport‐related genes, *ZmNPF2.9b* and *ZmNPF3.1*, in the *pUbi‐ZmMs1* (#1) line and WT by RT‐qPCR analysis. **(J**, **K)** Total GA **(J)** and ABA **(K)** contents in WT and *pUbi‐ZmMs1* (#1) line stems. **(L)** ChIP‐seq peaks showing the enrichment of ZmMS1 in promoters of *ZmNPF2.9b* and *ZmNPF3.1*. **(M)** EMSA analysis of ZmMS1–LOB binding to the promoters of *ZmNPF2.9b* and *ZmNPF3.1*. **(N)** Transcriptional repression of *ZmNPF2.9b* and *ZmNPF3.1* by ZmMS1. **(O)** Schematic diagram of overexpression of *ZmMs1* affecting phytohormone GA and ABA contents by regulating GA and ABA biosynthesis, degradation, transport and signaling‐related genes. Data in **(B**, **E**–**G**, **I**–**K**, **N)** represent means ± *SD*. *n* = 20 for **(B)**; *n* = 3 for **(E**–**G**, **I**–**K**, **N)**, and asterisks indicate significant differences (*P < 0.05, **P < 0.01, and ***P < 0.001). A two‐tailed Student's *t*‐test was used to determine *P* values.

We next performed RNA sequencing using the fifth internode tissues of WT and *pUbi‐ZmMs1* (#1) at the jointing stage to identify genes associated with the dwarf phenotype (Figure S10A; Dataset S8). As ZmMS1 is a transcriptional repressor (Hou et al., 2023), GO analysis of the significantly downregulated genes showed enrichment in the carbohydrate metabolic process (Figure S10B). Notably, multiple genes involved in phytohormone homeostasis, particularly gibberellic acid (GA) and abscisic acid (ABA), were differentially expressed between WT and *pUbi‐ZmMs1* (#1). In *pUbi‐ZmMs1* (#1), genes involved in GA degradation and negative regulation of GA signaling were upregulated, while those for GA biosynthesis and transport were downregulated. On the contrary, ABA biosynthesis and positive regulation of ABA signaling genes were upregulated, while a negative regulator of ABA signaling, *ZmPP2C16*, was downregulated (Figure 4H; Table S1). RT‐qPCR analysis validated these expression changes (Figures 4I, S10C, D). Consistent with the expression changes of these genes, the total GA content was significantly lower in *pUbi‐ZmMs1* (#1) than that in WT (Figure 4J). Consistently, most of the major components of GA were significantly decreased in *pUbi‐ZmMs1* (#1) (Figure S10E). In contrast, the total ABA content was markedly increased in *pUbi‐ZmMs1* (#1) compared to WT (Figure 4K; Dataset S9).

ChIP‐seq analysis showed that ZmMS1 enriched the promoter fragments of two GA transport genes: *ZmNPF2.9b* and *ZmNPF3.1* (Figure 4L). EMSA and TDLR assays confirmed that ZmMS1 binds to their promoter sequences containing the ZmMS1‐binding motifs and represses their promoter activities (Figure 4M, N). These results indicate that ZmMS1 directly represses these GA transporter genes in stems. Taken together, the constitutive ectopic overexpression of *ZmMs1* alters expression of phytohormone‐related genes, especially GA and ABA metabolism‐related genes, accompanied by decreased GA and increased ABA levels, which likely contributes to the reduction in maize plant height (Figure 4O).

### Ectopic overexpression of *ZmMs1* produces small and male‐sterile pollen grains

In addition to dwarfism, the *pUbi‐ZmMs1* line produced pollen grains with variable size (Figure 5A). The larger pollen grains in *pUbi‐ZmMs1* (#1) were comparable to those of the WT, but the diameters of smaller pollen grains were significantly reduced (Figure 5B). *ZmMs1* was expressed from S8b to S10, with a peak at S9 in WT anthers, while it was continuously expressed from S5 to S12 in *pUbi‐ZmMs1* (#1) anthers (Figure 5C), suggesting that the altered *ZmMs1* expression pattern underlies pollen size variation. Despite shorter anthers and some pollen grains being smaller than WT, no obvious morphological defects in anther cuticle, Ubish bodies, or pollen development were observed in *pUbi‐ZmMs1* (#1) (Figures 5D, S11). Approximately 50% of pollen grains in *pUbi‐ZmMs1* (#1) anthers were smaller (Figures 5E, S12A), and these smaller pollen grains failed to germinate *in vitro* (Figure 5F), resulting in an overall pollen germination rate of about 50% (Figure 5G). Transformation of the *pUbi‐ZmMs1* cassette into the *ms1‐alb* mutant did not restore maize fertility. However, unlike *ms1‐alb*, the three *pUbi‐ZmMs1* lines with the *ms1‐alb*/*ms1‐alb* genotype that shed pollen showed pollen size variation even though all pollen grains were unstained by I_2_‐KI (Figure 5H, I). Since the percentage of smaller pollen grains in the *pUbi‐ZmMs1* (#1) line was approximately 50%, which suggests that this line has one *pUbi‐ZmMs1* insertion copy, we self‐crossed the dwarf plant of this line for several generations, and the selfed progeny of *pUbi‐ZmMs1* (#1) always showed normal and dwarf plants with segregation ratios of around 1:1 (Figure S12B). This suggests that pollen grains carrying the *pUbi‐ZmMs1* expression cassette are male‐sterile and non‐transmissible.

**Figure 5:**
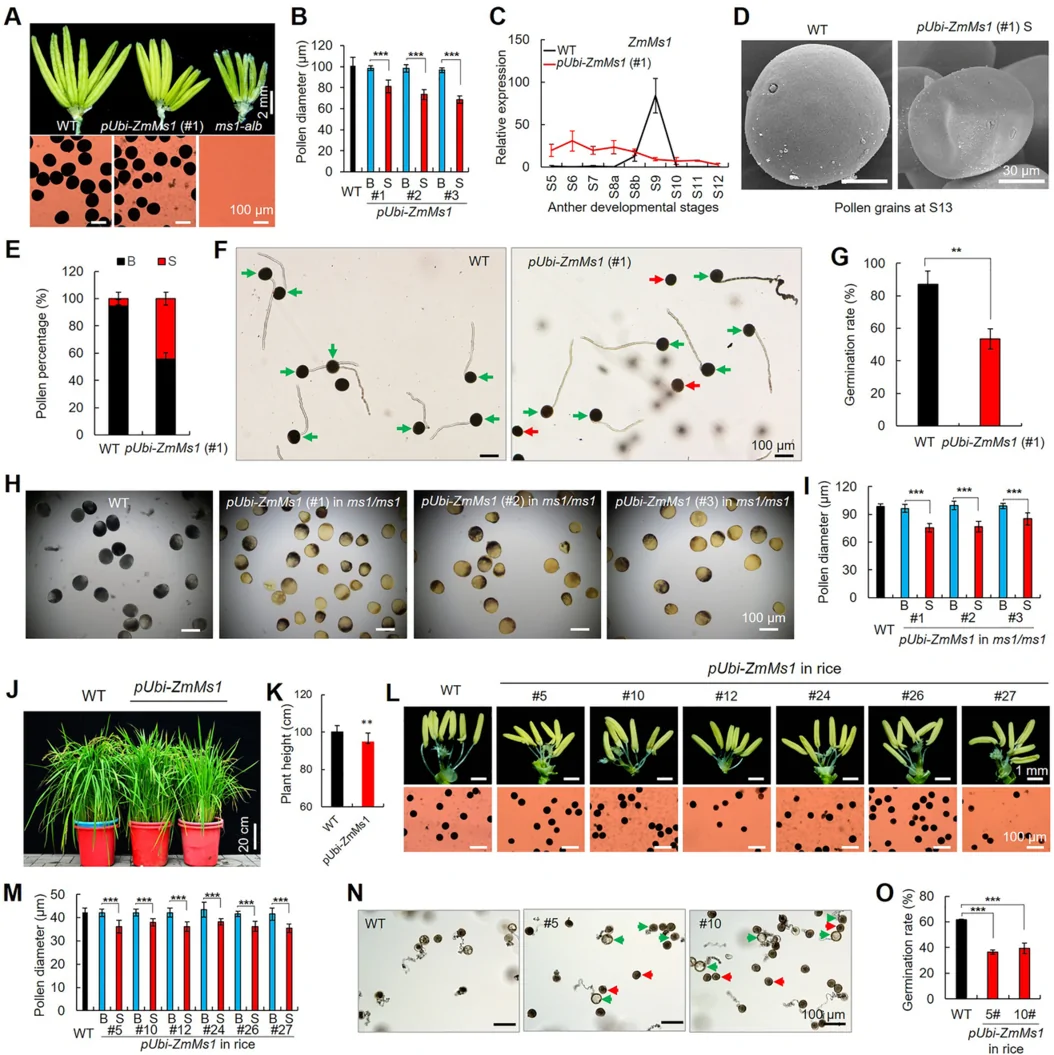
**Ectopic overexpression of** ***ZmMs1*** **produces small pollen grains with male sterility** **(A)** Anthers and pollen grains (stained by I_2_‐KI) of WT (B73), the *pUbi‐ZmMs1* (#1) line (*ZmMs1*/*ZmMs1* // *pUbi‐ZmMs1*/−), and *ms1‐alb*. **(B)** Quantitative measurements of pollen diameters of WT and *pUbi‐ZmMs1* lines. B and S represent big (diameter > 90 μm) and small pollen grains (diameter ≤ 90 μm), respectively. **(C)** *ZmMs1* expression in WT and *pUbi‐ZmMs1* (#1) anthers from S5 to S12 by RT‐qPCR analysis. **(D)** Comparison of WT and *pUbi‐ZmMs1* (#1) pollen grains based on SEM observation at S13. **(E)** Proportions of small pollen grains in WT and the *pUbi‐ZmMs1* (#1) line. B and S represent big (diameter > 90 μm) and small pollen grains (diameter ≤ 90 μm), respectively. **(F)** *In vitro* pollen germination assay of WT and the *pUbi‐ZmMs1* (#1) line. Green arrows indicate germinated big pollen grains and red arrows indicate small pollen grains that cannot germinate. **(G)** Pollen germination rates of WT and the *pUbi‐ZmMs1* (#1) line. **(H)** Pollen grains (stained by I_2_‐KI) of WT (B73) and the *pUbi‐ZmMs1* (#1) line in the *ms1‐alb* background (*ms1‐alb*/*ms1‐alb* // *pUbi‐ZmMs1*/*−*). **(I)** Quantitative measurements of pollen diameters of WT and the *pUbi‐ZmMs1* (#1) line in the *ms1‐alb* background. B and S represent big (diameter > 90 μm) and small pollen grains (diameter ≤ 90 μm), respectively. **(J)** Whole‐plant phenotype of WT (Nipponbare) and *pUbi‐ZmMs1* transgenic rice lines. **(K)** Quantitative measurements of plant height of WT and *pUbi‐ZmMs1* transgenic rice lines. **(L)** Anthers and pollen grains (stained by I_2_‐KI) of WT and six *pUbi‐ZmMs1* transgenic rice lines. **(M)** Quantitative measurements of pollen diameters of WT and six *pUbi‐ZmMs1* transgenic rice lines. B and S represent big (diameter > 40 μm) and small pollen grains (diameter ≤ 40 μm), respectively. **(N)** *In vitro* pollen germination assay of WT and two *pUbi‐ZmMs1* transgenic rice lines. Green arrows indicate germinated big pollen grains and red arrows indicate small pollen grains that cannot germinate. **(O)** Pollen germination rates of WT and *pUbi‐ZmMs1* transgenic rice lines. Data in **(B**, **C**, **E**, **G**, **I**, **K**, **M**, **O)** represent means ± *SD*. *n* > 20 for **(B**, **G**, **I**, **M)**; *n* = 3 for **(C**, **E)**; *n* = 6 for **(K)**, and asterisks indicate significant differences (**P < 0.01 and ***P < 0.001). A two‐tailed Student's *t*‐test was used to determine *P* values.

To investigate whether overexpressing *ZmMs1* causes similar abnormalities in rice, we transformed the *pUbi‐ZmMs1* plasmid into rice (Nipponbare). All six transgenic rice lines showed decreased plant height compared to WT (Figure 5J, K), and all the transgenic lines could harvest seeds, suggesting that they were male‐fertile. However, similar to maize, *pUbi‐ZmMs1* transgenic rice lines produced smaller pollen grains compared to WT (Figure 5L, M), which also failed to germinate *in vitro* (Figure 5N, O). Together, the constitutive overexpression of *ZmMs1* reduces plant height and results in the production of small and sterile pollen grains in both maize and rice.

### Development of a multi‐control sterility system in maize based on the *ms1‐alb* mutant and the *ZmMs1* gene

A key requirement for hybrid seed production is stable and complete sterility of female parent lines across diverse genetic backgrounds. The *ms1‐alb* male‐sterile gene showed stable and complete male sterility under field conditions across 547 maize genetic backgrounds (Figure S13; Datasets S10, S11), highlighting its breeding potential. We designed an MCS strategy for maize breeding utilization of the *ms1‐alb* gene (Figure S14A). An MCS vector of *pMCS0107* containing five functional modules (*pZmMs1‐ZmMs1*, *pZm13‐Dam*, *pPG47‐ZmAA*, *pLTP2‐mCherry*, and *p35S‐Bar*) was constructed (Figure 6A), transformed into maize Hi II (Figure 6B, C), and introgressed into the *ms1‐alb* mutant via marker‐assisted selection (Figure S14B–E). The resulting eight *pMCS0107* transgenic maintainer lines showed male fertility with ~50% transgenic inviable pollen grains, leading to ~50% normal yellow color male‐sterility seeds and ~50% transgenic red‐fluorescent seeds in self‐pollinated plants (Figures 6B, C, S14B–E). Transgene transmission rates of the eight T_1_ generation maize plants measured through the transgenic pollen test ranged from 0 to 0.29‱, suggesting that the *pMCS0107*‐based MCS system can produce non‐transgenic maize male‐sterility lines. The color distinction enables efficient sorting of transgenic seeds for propagating maintainer lines and non‐transgenic seeds for propagating male‐sterility lines as female parents for hybrid breeding and seed production in maize (Figure S14).

**Figure 6:**
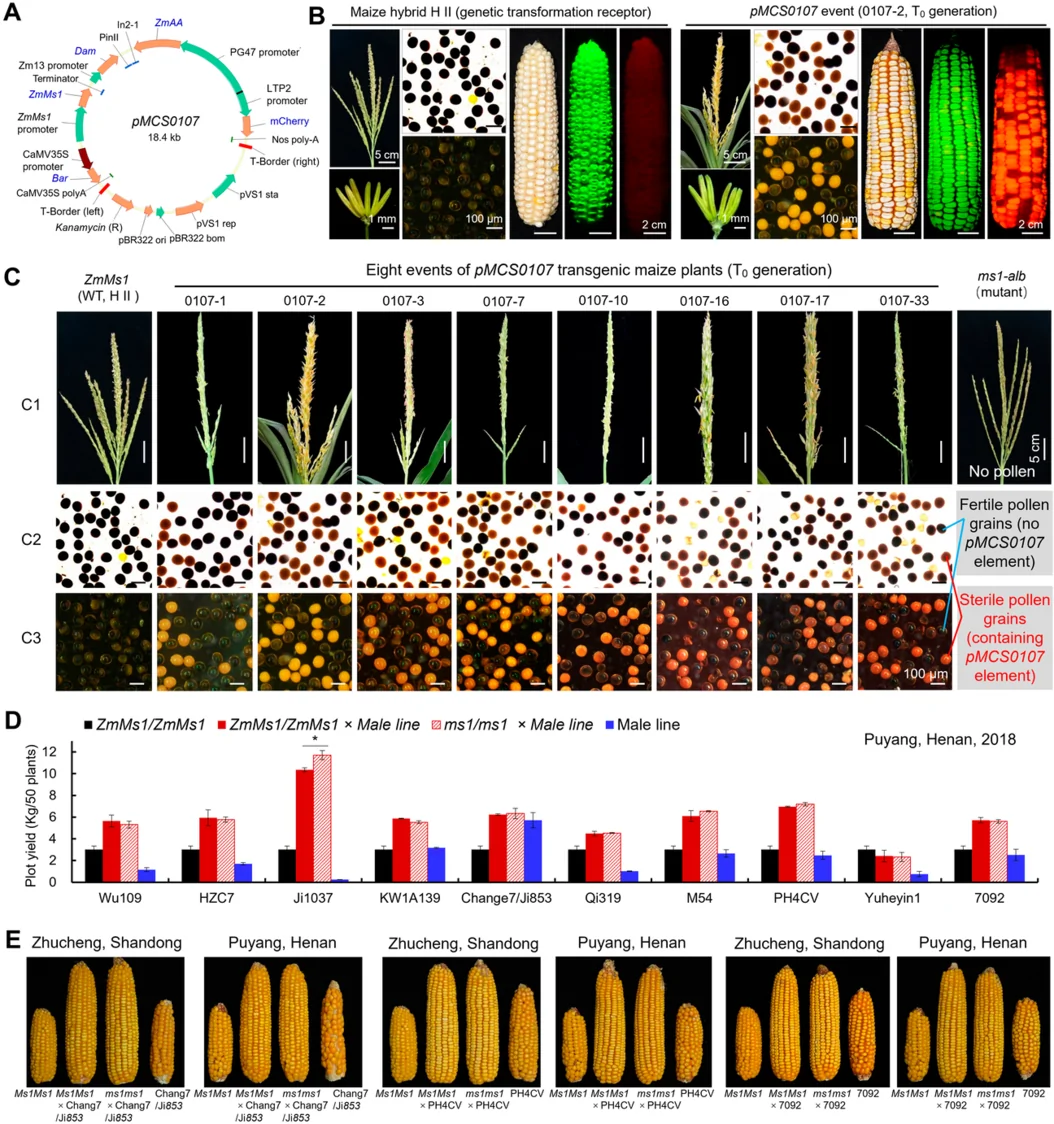
**Development of an MCS system in maize based on the** ***ms1‐alb*** **mutant and the** ***ZmMs1*** **gene for creating male‐sterility lines and their breeding application test** **(A)** The MCS construct (*pMCS0107*) contains five functional modules: *pZmMs1*‐*ZmMs1*, *pZm13‐Dam*, *pPG47*‐*ZmAA*, *pLTP2‐mCherry*, and *pCaMV35S‐Ba*r. **(B)** Tassels, anthers, pollen grains (stained by I_2_‐KI), and ears of WT and a representative *pMCS0107* transgenic event (0107‐2) at the T_0_ generation. The photographs of ears were taken under bright light and green excitation light with red fluorescence filter I (GREEN.L, China) and red fluorescence filter II (NIGHTSEA, USA). **(C)** Tassels (C1) and pollen grains (stained by I_2_‐KI) (C2 and C3) of WT, the *ms1‐alb* mutant, and eight transgenic MCS maintainer lines at the T_0_ generation. **(D)** Analysis of plot yield of ten representative hybrid combination pairs from the crosses between the male‐sterile *ms1‐alb* line or WT and ten maize male lines in Puyang, Henan province, China. Data represent means ± *SD* of 50 plants per cross with two replicates (*P < 0.05), and a two‐tailed Student's *t*‐test was used to determine *P* values. **(E)** Ear comparison of three hybrid combination pairs derived from WT and the *ms1* mutant being crossed with three different male lines planted at the experimental stations in Zhucheng, Shandong province, and Puyang, Henan province, China.

For breeding application, male*‐*sterility genes should not be linked to undesirable traits or penalize important agronomic traits such as plant growth or yield. To assess whether the *ms1‐alb* mutation affects phenotypes of agronomic traits, particularly grain yield, we selected 30 elite inbred lines as male parents to simultaneously cross with *ZmMs1* and *ms1‐alb* homozygous plants. The resulting F_1_ hybrids and their corresponding parental lines were cultivated in two different experimental locations: Zhucheng, Shandong province, and Puyang, Henan province. Seventeen agronomic traits (Dataset S12), including plot yield, plant height, *etc*., were evaluated to compare heterosis and field performance among these hybrid combinations. Regarding plot yield, all hybrid combinations showed substantially higher plot yield than their parents. No significant difference (*P* < 0.05) in plot yield was detected among the 30 hybrid pairs with *ms1‐alb* vs WT female parents, except that the *ms1* × Ji1037 hybrid showed a slightly higher yield than the WT × Ji1037 hybrid in the Puyang environment, and the *ms1* × Yuheyin1 hybrid showed a slightly lower yield than the WT × Yuheyin1 hybrid in the Zhucheng environment (Figures 6D, E, S15–S18). For the other important agronomic trait, plant height, significant differences between hybrids with *ZmMs1* vs *ms1‐alb* as the female parents were observed in several combinations, but only in one environment (Figure S18B, C; Dataset S12). The direction of change appeared completely random across hybrid pairs. Collectively, the *ms1‐alb* gene had no obvious adverse effects on heterosis or field performance, and its male sterility was stable and complete across various genetic backgrounds, suggesting that the *ZmMs1* gene and its *ms1‐alb* mutant are applicable for hybrid breeding and seed production in maize.

### Development of the *p5126‐ZmMs1‐*based dominant genic male sterility system in maize

Because the *p5126‐ZmMs1* transgenic maize plants display DGMS without viable pollen grains (Figure 1A), an improved *p5126‐ZmMs1D* vector by adding a red fluorescence seed‐sorting module (*pLTP2‐DsRed2*) was constructed (Figure 7A) to develop a DGMS system for hybrid maize breeding. The construct was transformed into immature embryos of maize Hi II, and four independent transgenic events (*p5126‐ZmMs1D*‐#1 to #4) were obtained and maintained through pollination with maize B73 pollen (Figures 7B, S19A). One event *p5126‐ZmMs1D* (#1), pollinated by B73 pollen, produced ~50% transgenic fluorescent DGMS seeds and ~50% non‐transgenic normal color seeds, which can be color‐sorted efficiently (Figures 7C, S19B, C). DGMS sterility of *p5126‐ZmMs1D* (#1) was tested by crossing with 379 genetically diverse lines (Dataset S13). All F_1_ hybrid seeds contained ~50% DGMS seeds and ~50% male‐fertile seeds. After color sorting, the red fluorescent F_1_ seeds carrying the *p5126‐ZmMs1D* transgenic element showed stable and complete dominant male sterility (Figures S20, S21), indicating that the *p5126‐ZmMs1D‐*driven DGMS system is suitable for practical hybrid maize breeding.

**Figure 7:**
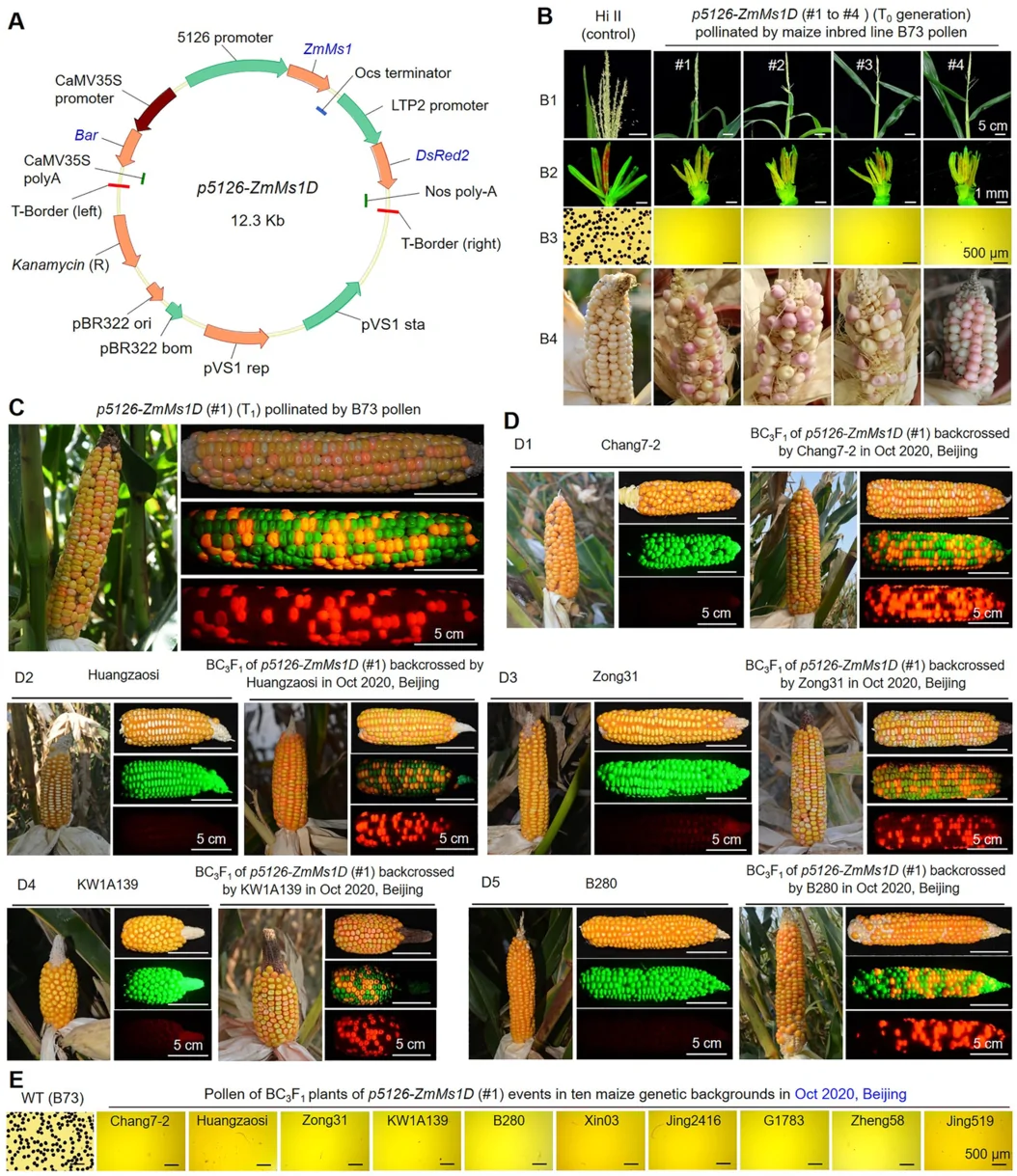
**Development of a** ***p5126‐ZmMs1D*** **‐based DGMS system for maize breeding and hybrid seed production** **(A)** The DGMS construct (*p5126‐ZmMs1D*) contains three functional modules: *p5126‐ZmMs1*, *pLTP2‐DsRed2*, and *pCaMV35S‐Bar*. **(B)** Phenotypic characterization of WT (Hi II) and four representative maize transgenic events (*p5126‐Ms1D* #1 to #4) at the T_0_ generation. Tassels (b1); anthers (b2); pollen grains (b3) stained by 1% I_2_‐KI; and ears (b4) pollinated by B73 pollen. **(C)** Ear phenotypes of a representative maize transgenic event *p5126‐ZmMs1D* (#1) at the T_1_ generation, under bright light and green excitation light with red fluorescence filter I (GREEN.L, China) and red fluorescence filter II (NIGHTSEA, USA). **(D)** The BC_3_F_1_ ears of *p5126‐ZmMs1D* (#1) backcrossed by five maize male lines were photographed with a Canon EOS 700D digital camera (Canon, Japan) in the field under bright light, and green excitation light with red fluorescence filter I (GREEN.L, China) and red fluorescence filter II (NIGHTSEA, USA). **(E)** Pollen grains (stained by I_2_‐KI) of WT (B73) and *p5126‐ZmMs1D* (#1) transgenic event in ten maize genetic backgrounds at the BC_3_F_1_ generation.

We then crossed and backcrossed *p5126‐ZmMs1D* (#1) by ten maize inbred lines as the recurrent parents to obtain the BC_1_F_1_ generations (Figure S22). After being backcrossed for two and three generations, the ten BC_2_F_1_ and BC_3_F_1_ lines of *p5126‐ZmMs1D* (#1) showed stable and complete dominant male sterility (Figures 7D, S23), indicating that the *p5126‐ZmMs1D* transgenic element is genetically transmitted only through female gametes. In application, the DGMS and male‐fertility seeds of F_1_ hybrid can be randomly mixed and planted together, or the non‐transgenic F_1_ hybrid seeds can be selectively sorted out for production based on the region's GMO regulatory policy (Figure S19C). In addition, our previous study showed that rice *p5126‐ZmMs1* transgenic lines also displayed dominant male sterility (Hou et al., 2023). Collectively, the *p5126‐ZmMs1D*‐based DGMS system could be a new addition to the maize and rice breeding toolboxes.

## DISCUSSION

This study elucidates the multifaceted roles of ZmMS1 as a central regulator in integrating ROS signaling and lipid metabolism to coordinate anther development and exine formation, and ectopic overexpression of *ZmMs1* remodels plant architecture, resulting in decreased plant height by intervening phytohormone homeostasis. The ZmMS1‐driven feedback repression loop controls anther ROS homeostasis by repressing ROS‐related genes, thereby ensuring timely tapetal PCD, and balances lipidic allocation between anther cuticle and pollen exine. Conversely, ZmMS1 activity is modulated by the redox state. Mutation of *ZmMs1* results in lower ROS levels at S9 to S9–10 and thus delayed tapetal PCD, and lipidic partitioning shows an altered allocation toward exine formation in the *ms1‐alb* mutant, leading to recessive male sterility. Precocious expression of *ZmMs1* causes excess ROS accumulation at S6 to S8, which triggers earlier tapetal PCD and impairs total lipid metabolism in anther tapetum, causing dominant male sterility (Figure 8A). The stable and complete sterility of both loss of function and precocious expression of *ZmMs1* in various genetic backgrounds enables the development of MCS and DGMS systems for creating male‐sterility lines for maize breeding and hybrid seed production (Figure 8B). Additionally, the constitutive ectopic overexpression of *ZmMs1* under the maize *ubiquitin* promoter reduces maize plant height by influencing phytohormone levels in stems and induces approximately 50% of male‐sterile and small pollen grains, offering potential for dwarf breeding and yield improvement in maize (Figure 8C, D).

**Figure 8:**
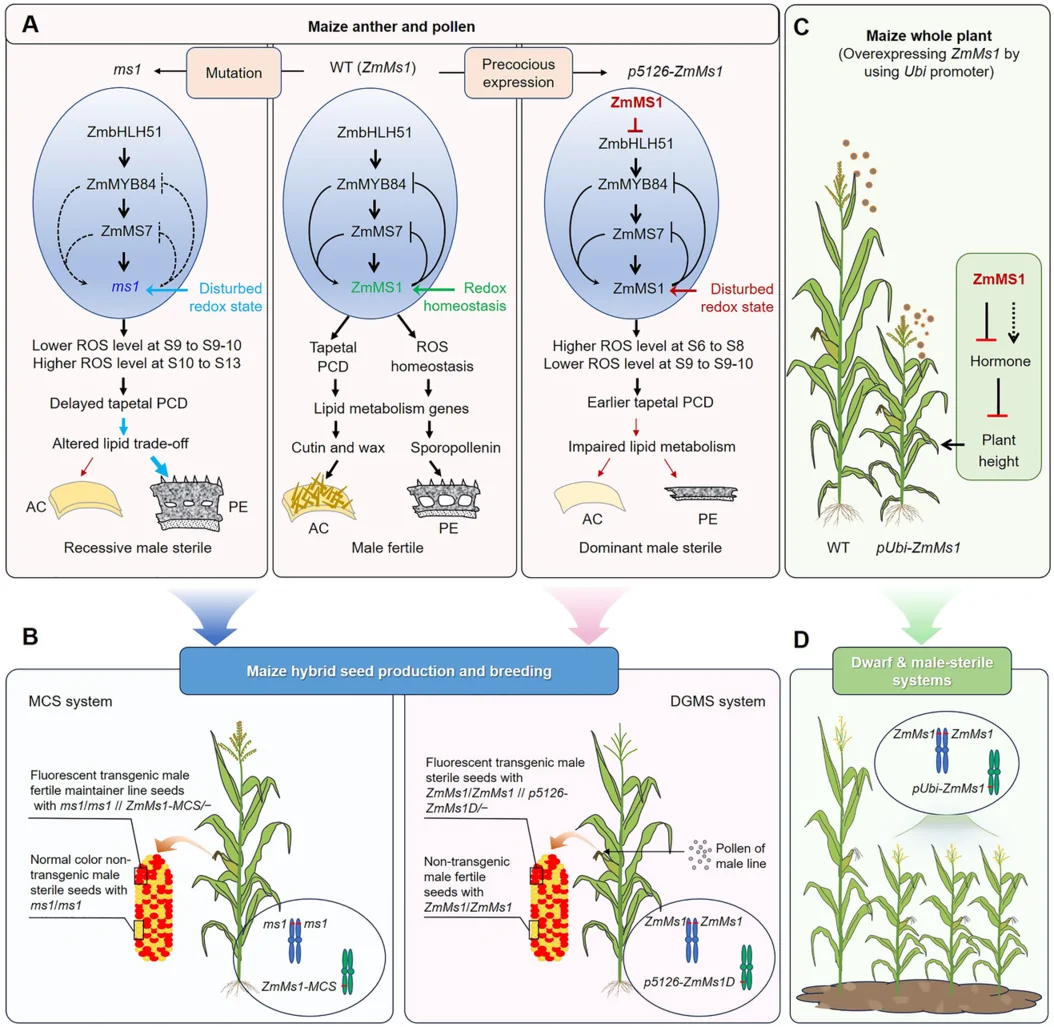
**Pleiotropic effects of** ***ZmMs1*** **and its potential breeding applications** **(A)** Function of *ZmMs1* in controlling anther and pollen development. Endogenous expression of *ZmMs1* in maize anthers controls male fertility by regulating ROS‐ and lipid metabolism‐related genes through a potential feedback repression regulation mechanism. Conversely, the DNA‐binding capacity of ZmMS1 is affected by redox state. Loss of *ZmMs1* (*ms1‐alb*) results in reduced ROS levels at S9 to S9–10, and thus delayed tapetal PCD and lipid partitioning, showing an altered allocation between anther cuticle and pollen exine, leading to male sterility. Precocious expression of *ZmMs1* driven by an anther‐specific promoter *p5126* causes excess ROS accumulation at S6 to S8, which triggers earlier tapetal PCD and impairs lipid metabolism, resulting in dominant male sterility. **(B)** The developed MCS and DGMS systems based on the sterility phenotypes in **(A)** for creating male‐sterility lines in maize hybrid seed production and breeding. **(C)** Effects of *ZmMs1* in decreasing plant height. Ectopic overexpression of *ZmMs1* under the maize ubiquitin promoter reduces maize plant height by modulating stem hormone levels. **(D)** Ectopic overexpression of *ZmMs1* offers potential for dwarf & male‐sterile breeding to improve lodging resistance and yield.

An intriguing finding is the dual relationship between ZmMS1 and anther redox status. ZmMS1 modulates ROS‐related genes to regulate a sudden massive production of ROS at S9 and S9–10 (Figure 1C, D) for triggering tapetal PCD in a timely manner (Figure S1), but its DNA‐binding activity is sensitive to oxidation status via four cysteines in its LOB domain (Figure 2E–H). Although we cannot dissect precise ROS levels in living tapetal cells and their influence on ZmMS1 activity *in vivo*, given that endogenous expression of *ZmMs1* displays a very narrow window with a peak at S9 (Hou et al., 2023), this suggests a potential redox‐dependent feedback model. The ZmMS1 dimers under reduction conditions are active and repress ROS‐scavenging genes, thereby allowing ROS to accumulate to a threshold that triggers tapetal PCD. Subsequently, the rising ROS in anthers may oxidize ZmMS1, thereby impairing its binding capacity, which releases the repression of ZmMS1 on ROS‐scavenging genes, helping to clean ROS and prevent excessive oxidative damage after PCD initiation. Such a redox‐sensitive switch could ensure that the repressive action of ZmMS1 is precisely confined in time, providing a robust yet transient signal for developmental progression.

The other intriguing finding is that ZmMS1 orchestrates the lipid allocation in tapetum by bifurcating into two pathways for pollen exine and anther cuticle development (Figure 3I). The ZmMS1‐mediated feedback repression loop and its key components directly activate or repress sporopollenin biosynthesis genes to control the precise amount of sporopollenin for normal exine formation (Hou et al., 2023) and indirectly influence cutin‐ and wax‐related genes to promote cuticle formation (Figures 3C, S6D). In the *ms1‐alb* mutant, the released repression of ZmMS1 induces overinvestment in sporopollenin and leads to thicker exine at the expense of cuticle lipids with a smooth anther surface (Figure 3A, B, D). When *ZmMs1* is precociously expressed (*p5126‐ZmMs1*) in anthers, the tapetal PCD occurs early (Hou et al., 2023), thereby precociously shutting down the lipid metabolism pathways and leading to deficiencies in both exine and cuticle (Figure 3F–H). Given that *ZmMYB84* and *ZmPKSB* are the target genes repressed by ZmMS1 (Liu et al., 2022; Hou et al., 2023), the opposite phenotypes in *myb84* and *pksb* mutants, with enhanced anther cuticle but impaired pollen exine, complement this model (Figures 3A, D, S6A, B). This establishes a coherent transcriptional network where ZmMS1 fine‐tunes the lipid flux, ensuring the simultaneous formation of a robust pollen wall and a functional anther envelope.

Beyond its anther‐specific roles, the constitutive ectopic overexpression of *ZmMs1* (*pUbi‐ZmMs1*) unveils pleiotropic effects. It causes significant dwarfism in both maize and rice with transcriptional reprogramming of GA and ABA signaling pathways, accompanied by decreased GA and elevated ABA levels in stems, which likely contributes to cell division and elongation inhibition. This suggests that ZmMS1 and/or its targets can intervene in core hormonal pathways governing plant growth. Notably, the constitutive overexpression of *ZmMs1* also leads to the production of ~50% small and sterile pollen grains in both maize and rice. The pleiotropic effects of *pUbi‐ZmMs1* on plant dwarfism and male sterility provide an opportunity for crop breeding and hybrid seed production to enhance grain yield (Ansari et al., 2017; Zheng et al., 2025). Thus, *ZmMs1* represents a potential genetic resource for developing a dwarf and male‐sterile system in cereals.

In conclusion, ZmMS1 is a multi‐function redox‐sensitive transcription repressor coordinating key anther developmental transitions. Its constitutive ectopic overexpression shows pleiotropic effects on plant architecture and male fertility. Although the DGMS system faces regulatory challenges in GMO‐restricted regions, and the MCS system still shows a low but detectable rate of transgenic pollen transmission, the few seeds yielded by such transgenic pollen in the MCS system could be screened out by seed fluorescence in stringent regions. Nevertheless, the *ZmMs1‐*mediated MCS and DGMS systems provide applicable, efficient, and flexible tools for maize hybrid breeding and hybrid seed production. In addition, the demonstrated efficacy of the *p5126‐ZmMs1* DGMS (Hou et al., 2023) and the dwarf and male‐sterile phenotypes of *pUbi‐ZmMs1* transgenic rice (Figure 5J–O) suggest that a potential DMS system could also be developed across plant species.

## MATERIALS AND METHODS

### Plant materials and growth conditions

Plant materials used in this study were obtained as follows: Maize seeds of the *ms1‐alb* mutant, its WT sibling, and the Hi II hybrid parents (Hi II‐A and Hi II‐B) were obtained from the Maize Genetics Cooperation Stock Center, the *myb84* and *pksb* mutants were from our laboratory (Jiang et al., 2021a; Liu et al., 2022), all other maize inbred lines were from the School of Advanced Agricultural Sciences, University of Science and Technology Beijing (USTB), and rice seeds of the Nipponbare cultivar were from WIMIbio (http://www.wimibio.com/).

Maize hybrid Hi II and rice cultivar Nipponbare were used for genetic transformation. The resulting transgenic lines included, for maize, the previously reported line *p5126‐ZmMs1* (#1)(Hou et al., 2023) and the newly generated lines: *pUbi‐ZmMs1* #1–#5, eight *pMCS0107* events, and *p5126‐Ms1D* #1–#4, and for rice, six *pUbi‐ZmMs1* lines (#5, #10, #12, #24, #26, and #27).

Whole maize and rice plants (either field‐grown or transplanted from the field into pots), maize tassels, ears, and aboveground internodes were photographed using a Canon EOS 700D digital camera (Canon, Japan). For detailed morphological observation, anthers were examined under an SZX2‐ILLB stereomicroscope (Olympus, Japan), and pollen grains stained with 1% I_2_‐KI were observed using a BX‐53F microscope (Olympus, Japan).

T_0_ transgenic maize and rice plants were cultivated in a greenhouse in Beijing (40°03′N, 116°37′E) under controlled long‐day conditions (16 h light/8 h dark, 26°C/22°C). All other plants were grown under natural conditions at USTB experimental stations located in Beijing (40°05′N, 116°37′E), Sanya in Hainan Province (18°37′N, 109°21′E), Puyang in Henan Province (35°45′N, 115°01′E), and Zhucheng in Shandong Province (35°59′N, 119°24′E).

### Plasmid construction and plant transformation

The MCS construct was assembled by inserting designed expression modules into the backbone vector pCAMBIA3301 to generate the plasmid *pMCS0107* as previously described (Zhang et al., 2018). The coding sequence of ZmMs1 was amplified from B73 genomic DNA using primers Ms1‑F1 and Ms1‑R1. The PCR product was cloned into the pEASY‑T5 vector to generate the recombinant plasmid *pTZmMs1*. The *ZmMs1* CDS was then re‐amplified from this plasmid using primers Ms1‑F2 and Ms1‑R2, which incorporated restriction sites. The resulting fragment was digested with *BamH*I. For vector construction, the hygromycin resistance gene (*Hpt*) in the *pCXUN* vector was replaced with the *Bar* gene (Chen et al., 2009), generating the modified vector pBCXUN. The pBCXUN vector was digested with *Sma*I and *BamH*I. The digested *ZmMs1* fragment was ligated into the linearized *pBCXUN* to create the final expression construct, *pUbi‑ZmMs1*. Next, the *p5126‐ZmMs1‐Ocs* expression cassette from *p5126‐ZmMs1* (Hou et al., 2023) was introduced into the *pCLD* vector (Zhang et al., 2018) via *Hin*dIII and *EcoR*I restriction sites, resulting in the plasmid *p5126‐ZmMs1D*. The resulting constructs were genetically transformed into maize or rice as previously described (An et al., 2020). All primers used for plasmid construction are listed in Table S2.

### Tapetal cell DAPI staining

Anthers of WT, *ms1‐alb*, and *p5126‐ZmMs1* (#1) were embedded in paraffin (Sigma, USA) and sectioned with a microtome. The sections were fixed and stained by a 0.5 μg/mL 4’,6‐diamidino‐2‐phenylindole (DAPI) solution and imaged as previously described (Hou et al., 2023).

### Cytological analysis and microscopy

For semi‐thin transverse sections, fresh anthers were fixed in FAA solution (5% formaldehyde, 6% acetic acid, and 45% ethanol; Coolaber, China). For scanning electron microscopy (SEM) and transmission electron microscopy (TEM), anthers were fixed overnight in 3% glutaraldehyde. All subsequent steps were carried out as previously described (An et al., 2019).

### Measurement of ROS levels

Fresh anthers of WT, *ms1‐alb*, and *p5126‐ZmMs1* (#1) were washed in PBS with 0.2% Tween‐20 for 5 min, followed by staining with H_2_DCF‐DA (Sigma–Aldrich) as described (Xie et al., 2014). After washing with PBS, samples were imaged on a Leica TCS‐SP8 confocal microscope. ImageJ software was used to quantify the relative fluorescence intensities of ROS.

### RNA‐seq analysis

The anther RNA‐seq data of *ms1‐alb*, *p5126‐ZmMs1* (#1), and their WT at different developmental stages were derived from our previous study (Hou et al., 2023). Raw reads are available in the NCBI Sequence Read Archive under BioProject PRJNA785726 or at Science Data Bank (https://doi.org/10.57760/sciencedb.09688).

For stem RNA‐seq, fresh fifth‐internode samples were collected at the jointing stage from WT and *pUbi‐ZmMs1* (#1) plants. Total RNA was extracted and sequenced on an Illumina HiSeq platform by Novogene. Data analysis was performed as described previously (An et al., 2020; Dataset S8). Briefly, clean reads were aligned to the B73 reference genome (AGPv4) using TopHat 2.0 with default parameters. Gene expression levels were calculated and normalized as RPKM (reads per kb per million mapped reads) based on the maize gene annotations (Ensembl release 37) using Rsubread and edgeR. The GO enrichment analysis was performed using the web tool agriGOv2 (http://systemsbiology.cau.edu.cn/agriGOv2/).

### Purification of recombinant proteins

The full‐length coding sequences of ZmMS1, its four variants (ZmMS1^C9A^, ZmMS1^C12A^, ZmMS1^C19A^, and ZmMS1^C23A^), ZmMYB84, and the LOB domain of ZmMS1 (ZmMS1‐LOB, amino acids 1–113) were cloned in‐frame into the *pMCSG7* vector, which provides an N‐terminal His‐MBP tag. The empty vector, expressing MBP alone, served as a control. After transforming the plasmids into *E. coli* BL21 (DE3), recombinant protein expression was induced with 0.2 mM IPTG at 16°C overnight. Proteins were purified from harvested cells using Ni‐charged resin (Bio‐Rad, #1560131), desalted with PD‐10 columns (GE Healthcare), confirmed by SDS‐PAGE, and stored at −70°C. Primers used here are listed in Table S2.

### ChIP‐seq analysis

ChIP‐seq data for ZmMS1 were obtained from our previous study (Hou et al., 2023). Experimental and analytical procedures followed the description therein.

### Electrophoretic mobility shift assay (EMSA)

EMSA was performed as described previously (An et al., 2020). Briefly, biotin‐labeled or 5‐FAM (5‐Carboxyfluorescein)‐labeled probes were generated by annealing sense (5’‐biotinylated) and antisense primers. Each 20 μL binding reaction contained 400 ng of desalted recombinant protein and 0.02 pmol (biotin‐labeled) or 1 pmol (5’‐FAM‐labeled) probe in 1× binding buffer (GS005, Beyotime). After incubation, complexes were separated on 6% native polyacrylamide gels and observed directly under a multifunctional imaging system (Tanon Chemi Dog Ultra) with a green light filter (5’‐FAM‐labeled) or blotted onto positively charged nylon membranes (biotin‐labeled). Specificity was tested by competition with excess unlabeled probe with an identical sequence or mutated probes (amounts specified in figures). Purified MBP was used as a negative control. Primers used here are listed in Table S2.

### Transient dual‐luciferase assay

Effector constructs were generated by cloning the coding sequences of target genes into the *35S‐Ω* vector. Reporter constructs were made by inserting promoter regions amplified from B73 genomic DNA into the *pEASY‐LUC* vector. The *proAtUbiquitin3:REN* plasmid was the internal control. Assays were performed in maize protoplasts as described (An et al., 2020), and the relative luciferase activity (LUC/REN) was measured. Primers used here are listed in Table S2.

### Protein structure prediction

The three‐dimensional structure of ZmMS1 was predicted using Alphafold3 (https://alphafoldserver.com/) and visualized using PyMOL (Schrödinger, LLC). Conserved residues and putative functional sites were identified through multiple sequence alignment with homologous proteins. To visualize potential metal‐binding sites, a zinc ion (Zn^2+^) binding was predicted using a web server (https://aideepmed.com/IonCom/) and incorporated into the model using PyMOL. The DNA duplex was modeled with a web tool (https://www.scfbio-iitd.res.in/software/drugdesign/bdna.jsp) and the protein–DNA docking was performed using HADDOCK (https://wenmr.science.uu.nl/haddock2.4/) as described (Chen et al., 2019). The protein–DNA complex structure was modified in PyMOL. The crystal structure of the wheat Ramosa2 LOB domain dimer was obtained from Protein Data Bank under the accession number: 5ly0 (https://www.rcsb.org/structure/5LY0). The LOB domain structure alignment was carried out in PyMOL.

### Yeast two‐hybrid (Y2H) assay

The coding sequence of ZmMS1–LOB was amplified and cloned into the *pGBKT7* and *pGADT7* vectors. The resulting *pGBKT7–ZmMS1–LOB* and *pGADT7–ZmMS1–LOB* plasmids were co‐transfected into the yeast strain Y2HGold and verified on selective media as described in the Matchmaker Gold Yeast Two‐Hybrid System (630489, Takara). Primers used here are listed in Table S2.

### Luciferase complementation assay

The ZmMS1–LOB coding sequence was cloned into the *pLYY01‐nLUC* and *pJG014‐cLUC* vectors. After transformation into *Agrobacterium tumefaciens* strain LBA4404, the bacteria were cultured to OD_600_ = 1.0 and resuspended in activation buffer (10 mM MgCl_2_, 10 mM MES, and 200 μM acetosyringone). Desired bacterial combinations were mixed in the dark for 3 h and co‐infiltrated into *N. benthamiana* leaves (Fujikawa and Kato, 2007). Luciferase activity was detected by spraying leaves with D‐luciferin (Promega, E1602) and imaged using a multifunctional imaging system (Tanon Chemi Dog Ultra). Primers used here are listed in Table S2.

### LC‐MS/MS analysis of disulfide bonds

Purified ZmMS1 protein treated with or without DTT was digested with pepsin, trypsin, endoproteinase Lys‐C, and Asp‐N, and the resulting peptides were analyzed by liquid chromatography‐tandem mass spectrometry (LC‐MS/MS) following the protocol described previously (Ni et al., 2013).

### BIAM labeling assay

BIAM labeling assays were performed as described previously (Tian et al., 2018). Specifically, 300 ng of purified MBP, MBP‐ZmMS1, and MBP‐ZmMS1‐LOB proteins were treated with 0, 0.2, 1, or 10 mM of H_2_O_2_ in PBS solution in a total of 40 μL for 15 min in darkness. The proteins were precipitated by incubation at −20°C for 20 min with one volume of acetone and collected by centrifugation at 6,000× *g* for 5 min. After three washes with precooled 50% acetone, the proteins were dissolved in 500 μL of labeling buffer containing 50 mM MES‐NaOH pH 6.5, 100 mM NaCl, 1% TritonX‐100, and 100 μM BIAM (AS‐60644, Anaspec), and incubated at room temperature in darkness for 1 h. The labeling reactions were terminated by the addition of β‐mercaptoethanol to a final concentration of 20 mM. The proteins were precipitated again with acetone as described above and dissolved in 50 μL of SDS sample buffer. Proteins labeled with BIAM were detected with HRP‐conjugated streptavidin (7075S, Cell Signaling). The MBP antibody (E8032S, NEB) was used to detect the total MBP, MBP–ZmMS1, or MBP–ZmMS1–LOB proteins.

### Analysis of anther cutin, wax, and internal lipids

Anthers from the WT, *ms1‐alb*, *myb84*, and *pksb* plants at stage S13 were collected and freeze‐dried. To precisely calculate the amount per unit surface area, the ratio of anther weight to surface area was determined and plotting of the anther dry weight against anther surface area was performed according to a previous study (An et al., 2020). Extraction and GC‐MS analysis of anther cutin, wax, and internal lipids were performed as previously described (Zhang et al., 2024).

### Quantitative real‐time PCR analysis

Total RNA was extracted from maize tissues using the TRIzol reagent (Invitrogen, USA) following the respective manufacturer's protocols. For cDNA synthesis, 1 μg of total RNA was reverse‐transcribed using the 5× All‐In‐One RT MasterMix (abm, Canada). Quantitative real‐time PCR (qPCR) was performed on a QuantStudio 5 Real‐Time PCR System (Applied Biosystems, USA) in triplicate, using TB Green Premix Ex Taq (Takara, Japan) according to the provided instructions. *ZmUBI2* (Zm00001d053838) was used as the internal reference gene for normalization. Primers used for qPCR are listed in Table S2. Data were analyzed using the 2^−ΔΔCt^ method, and quantitative results are described as means ± *SD*.

### Epidermal cell observation

The epidermis was carefully peeled from the fifth internode of maize plants at the jointing stage using fine forceps. Cell outlines were visualized under a Leica TCS‐SP8 confocal microscope by detecting the autofluorescence of lignin in the cell wall, excited with a 405 nm laser. Cell length was measured using ImageJ software.

### Measurement of GA and ABA content assay

The contents of gibberellins (GA) and abscisic acid (ABA) were quantified by MetWare (Wuhan, China; http://www.metware.cn/) following established UPLC‐ESI‐MS/MS protocols, with minor modifications (Pan et al., 2010; Šimura et al., 2018). In brief, fresh maize stem tissues were flash‐frozen in liquid nitrogen and homogenized. Approximately 50 mg of ground tissue was extracted with 1 mL of methanol/water/formic acid (15:4:1, v/v/v) containing 10 μL of internal standard (100 ng/mL). After centrifugation (12,000 rpm, 5 min, 4°C), the supernatant was dried, reconstituted in 80% methanol, and filtered (0.22 μm).

Chromatographic separation was performed on a Waters ACQUITY UPLC HSS T3 C18 column (100 × 2.1 mm, 1.8 μm) using water and acetonitrile (both with 0.04% acetic acid) with a linear gradient of 5%–95% B over 12 min at 0.35 mL/min. Detection was carried out on a QTRAP® 6500+ (Sciex, USA) in positive and negative ESI modes with MRM. Source parameters were ± 4,500–5,500 V, 550°C, and curtain gas 35 psi. Data were processed using Analyst 1.6.3 and MultiQuant 3.0.3. Hormone concentrations were calculated from calibration curves of authentic standards and normalized to internal standards. Significant changes were defined as fold change ≥ 2 or ≤ 0.5 (Cui et al., 2015; Dataset S9).

### Pollen germination assay

Mature pollen grains of maize (*Zea mays*) and rice (*Oryza sativa*) were collected from freshly dehisced anthers and immediately used for an *in vitro* germination assay. For maize, pollen grains were germinated on a solid medium containing 1 mM CaCl_2_, 1 mM Ca(NO_3_)_2_, 1 mM MgSO_4_, 0.01% (w/v) H_3_BO_3_, 18% (w/v) sucrose, and 0.8% (w/v) agar, adjusted to pH 7.0. After incubation at 28°C for 30 min, germination and pollen tube growth were observed and imaged using a BX‐53F microscope (Olympus, Japan). For rice, pollen grains were germinated on a medium containing 1.5 mM CaCl_2_, 1.5 mM Ca(NO_3_)_2_, 1 mM MgSO_4_, 0.01% (w/v) H_3_BO_3_, 12% (w/v) sucrose, and 0.5% (w/v) agar, adjusted to pH 6.0. After incubation at 26°C for 30 min, images were captured as in maize. The pollen germination rate was calculated as the percentage of germinated pollen grains relative to the total number of pollen grains observed. Each assay was repeated at least three times using independent biological samples.

### Fluorescent seed detection

Maize ears of *pMCS0107* transgenic events, *p5126‐ZmMs1D* transgenic lines, and their WT siblings were imaged under bright light and green excitation light with red fluorescence filter I (GREEN.L, China) and red fluorescence filter II (NIGHTSEA, USA).

### Transgene transmission rate analysis

To assess the transgene transmission rate via pollen, we pollinated the elite inbred line Zheng58 separately with pollen collected from eight transgenic MCS maintainer lines at the T_1_ generation. After harvest, red fluorescent seeds resulting from pollination with the corresponding transgenic pollen were manually counted under green excitation light using red fluorescence filter II (NIGHTSEA, USA).

### Analysis of male‐sterility stability of the *ms1‐alb* allele and *p5126‐Ms1D* (#1) under diverse genetic backgrounds

Male‐sterility stability analysis of the *ms1‐alb* allele and *p5126‐Ms1D* (#1) was performed according to our previous study (An et al., 2019; An et al., 2020). For *ms1‐alb* allele analysis, 547 maize inbred lines (Dataset S11) were used as female parents to cross with heterozygous *ZmMs1/ms1‐alb* plants in Beijing in 2017 summer. F_1_ progenies were grown in Sanya, Hainan province, in the winter of 2017. Heterozygous F_1_ progenies from each cross were identified by marker‐assisted selection and were self‐pollinated to produce F_2_ seeds. Fertility segregation ratios were investigated in all F_2_ populations grown in Beijing in the summer of 2018. Sterility was evaluated by collecting anthers from three sterile plants in each F_2_ population and examining pollen grains with 1% I_2_–KI stain. For *p5126‐Ms1D* (#1) analysis, 379 maize inbred lines (Dataset S13) were used as male parents to cross with *p5126‐Ms1D* (#1) plants in Beijing in the summer of 2019. Seeds of the resulting F_1_ progeny were selected based on color (red fluorescence) selection and were planted in Sanya, Hainan province, in the winter of 2019. The fluorescent F_1_ seeds were selected and grown in the next season and the sterility of these plants was evaluated as *ms1‐alb,* as described above.

### Evaluation of heterosis and agronomic traits in hybrids derived from *ZmMs1* and *ms1‐alb* homozygous female parents

To assess the impact of the *ms1‐alb* gene on heterosis and grain production, hybrid combinations were generated by crossing 30 diverse maize inbred lines, as male parents, with two distinct female parents: the *ms1‐alb* mutant and its homozygous WT sibling (*ZmMs1*/*ZmMs1*). The resulting 60 F_1_ hybrids and their parental lines were evaluated in duplicate field trials during the summer of 2018 at experimental stations in Zhucheng, Shandong Province, and Puyang, Henan Province, following standard maize field production practices. Seventeen agronomic traits, including plot yield and plant height, were investigated to compare heterosis and field performance between the two sets of hybrid combinations derived from the different female parents (Dataset S12).

### Statistical analysis

Statistical analysis of all the data was carried out using Microsoft Excel 2016 or GraphPad Prism 9 software. All values are presented as means ± *SD*. from at least three biological replicates and the number (*n*) of samples or experimental repeats; statistical significance determined with the unpaired two‐tailed Student's *t*‐test is indicated in the figure legends.

### Accession numbers

Maize stem RNA‐seq data of *pUbi‐ZmMs1* (#1) and WT generated in this study were deposited in the NCBI Sequence Read Archive (SRA) under the BioProject accession number PRJNA1406223. Maize anther RNA‐seq data of *ms1‐alb*, *p5126‐ZmMs1* and WT (accession number: PRJNA785726), and ChIP‐seq data of ZmMS1 (accession number: GSE220176) were retrieved from NCBI PRJNA785726 (Hou et al., 2023).

### Generative AI usage statement

During the preparation of this work, the authors utilized Deepseek (https://www.deepseek.com/) for the purpose of language editing. After using Deepseek, the authors carefully reviewed and revised the content, where necessary, and take full responsibility for the final version of the publication.

## CONFLICTS OF INTEREST

The authors declare no conflicts of interest.

## AUTHOR CONTRIBUTIONS

X.W. conceived and supervised this project and drafted all figures. Q.H. performed most of the molecular experiments and analyzed the data. X.A. and T.Z. performed cytological analysis and microscopy. X.A. and T.Y. performed H_2_DCFDA staining and analysis. Z.L. performed bioinformatic analysis. T.Z. and Bin.M assisted with RT‐qPCR experiments. L.Z. assisted with EMSA and TDLR experiments. Z.D., S.W., and Y.L. performed the field experiments. X.A, K.X., Y.J., and B.M. generated and detected the genetic materials. Q.H., X.A., and X.W. wrote and revised the manuscript. All authors read and approved the final manuscript.

## Supporting information

Additional Supporting Information may be found online in the supporting information tab for this article: http://onlinelibrary.wiley.com/doi/10.1111/jipb.70303/suppinfo

**Dataset S1.** Differentially expressed genes for pairwise comparisons at each developmental stage of WT anthers (corresponding to *ms1‐alb*) from developmental stage 5 to stage 12

**Dataset S2.** Differentially expressed genes for pairwise comparisons at each developmental stage of *ms1‐alb* anthers from developmental stage 5 to stage 12

**Dataset S3.** Differentially expressed genes for pairwise comparisons at each developmental stage between WT and *ms1‐alb* anthers from developmental stage 5 to stage 12

**Dataset S4.** Differentially expressed genes for pairwise comparisons at each developmental stage of WT anthers (corresponding to *p5126‐ZmMs1*) from developmental stage 5 to stage 12

**Dataset S5.** Differentially expressed genes for pairwise comparisons at each developmental stage of *p5126‐ZmMs1* anthers from developmental stage 5 to stage 12

**Dataset S6.** Differentially expressed genes for pairwise comparisons at each developmental stage between WT and *p5126‐ZmMs1* anthers from developmental stage 5 to stage 12

**Dataset S7.** Expression levels of 11 ROS‐scavenging genes in *ms1‐alb*, *p5126‐ZmMs1* (#1), and WT anthers based on RNA‐seq analysis

**Dataset S8.** Differentially expressed genes in *pUbi‐ZmMs1* (#1) identified by RNA sequencing

**Dataset S9.** Hormone content analysis in stems of *pUbi‐ZmMs1* (#1) and WT

**Dataset S10.** Stability assay of *ms1‐alb* male sterility through segregation ratios of fertile plants to sterile plants in the 547 F_2_ populations

**Dataset S11.** Genotypes of the 547 maize inbred lines used for examining male‐sterility stability of *ms1‐alb* in the maize 3072 SNP chip

**Dataset S12.** The 17 agronomic trait investigation for testing the effects of the *ms1‐alb* mutation on maize field production

**Dataset S13.** Genotypes of the 379 maize inbred lines for examining male‐sterility stability of *p5126‐ZmMs1* (#1) in the maize 3072 SNP chip

**Figure S1.** TEM analysis of anther wall layers in WT, *ms1‐alb,* and *p5126‐ZmMs1* (#1) lines from stages S8a to S11
**Figure S2.** Images showing ROS‐level differences between WT and *ms1‐alb* anthers from stages S6 to S13 detected by H_2_DCFDA staining
**Figure S3.** Images showing ROS‐level differences between WT and *p5126‐ZmMs1* (#1) anthers from stages S6 to S13 detected by H_2_DCFDA staining
**Figure S4.** Transcriptome analysis of *ms1‐alb* and *p5126‐ZmMs1* (#1) anthers from stages S5 to S12
**Figure S5.** Four cysteine residues in the LOB domain of ZmMS1 are essential for its DNA‐binding ability and to form disulfide bonds
**Figure S6.** Lipid contents and expression of cutin‐ and sporopollenin‐related genes in *ms1‐alb*, *myb84*, and *pksb* mutants and their corresponding WT anthers
**Figure S7.** GO functional annotation of DEGs in *ms1‐alb* and *p5126‐ZmMs1* (#1)
**Figure S8.** GO functional annotation of upregulated DEGs in *ms1‐alb* and downregulated DEGs in *p5126‐ZmMs1* (#1) by anther transcriptome analysis
**Figure S9.** Phenotypic characterization of *pUbi‐ZmMs1* (#1) transgenic maize plants
**Figure S10.** GA component contents and expression of GA‐ and ABA‐related genes in *pUbi‐ZmMs1* (#1) and WT stems
**Figure S11.** SEM and transverse section observations of anther and pollen development in WT and *pUbi‐ZmMs1*
**Figure S12.** Small pollen grains of *pUbi‐ZmMs1* (#1) cannot transmit to the next generation
**Figure S13.** Male‐sterility stability analysis of a homozygous *ms1‐alb* mutation after introgression into various maize genetic backgrounds
**Figure S14.** Strategy for developing an MCS system using the *ms1‐alb* mutant and the *ZmMs1* gene and maize phenotypes of four representative *pMCS0107* transgenic events (0107‐1, ‐3, ‐7, and ‐16)
**Figure S15.** Ear display in field plot experiments of two representative hybrid combination pairs derived from WT and the *ms1* mutant as female lines and Chang7/Ji853 and Dan598 as male lines
**Figure S16.** The 27 hybrid combination pairs derived from crossing WT and the *ms1* mutant with each of 27 different male lines were planted at the Zhucheng experimental station, Shandong province, China, for ear comparison
**Figure S17.** The 27 hybrid combination pairs derived from crossing WT and the *ms1* mutant with each of 27 different male lines were planted at the Puyang experimental station, Henan province, China, for ear comparison
**Figure S18.** Investigation of two representative agronomic traits for a heterosis assay of *ms1‐alb* mutation
**Figure S19.** Strategy for developing a *p5126‐ZmMs1D*‐based DGMS system
**Figure S20.** Dominant male‐sterility stability assay of the *p5126‐ZmMs1D* (#1) transgenic line after introgression into various maize genetic backgrounds
**Figure S21.** Pollen grains (stained by I_2_‐KI) of WT and a *p5126‐ZmMs1D* (#1) transgenic event under 95 genetic background lines
**Figure S22.** Phenotype of WT and a *p5126‐ZmMs1D* (#1) event in ten maize genetic backgrounds at the BC_1_F_1_ generation
**Figure S23.** Phenotypic comparison of the corresponding recurrent parent lines and a *p5126‐ZmMs1D* (#1) transgenic event in different maize genetic backgrounds at the BC_2_F_1_ and BC_3_F_1_ generations
**Table S1.** Expression changes of 16 GA and ABA metabolism‐related genes in *pUbi‐ZmMs1* (#1) and WT stems based on RNA‐seq analysis
**Table S2.** Oligonucleotides used in this study
