# Supplementary material for: ZmMS1 coordinates ROS homeostasis, lipid allocation, and male fertility for maize breeding applications
Source: J Integr Plant Biol. 2026 May 26;68(11):4030–51. doi: 10.1111/jipb.70303 (PMC13725604; doi:10.1111/jipb.70303)
Supplement: Supplementary file 1 [file JIPB-68-4030-s014.docx]

**Figure S1. TEM analysis of anther wall layers in WT, *ms1-alb,* and *p5126-ZmMs1* (#1) line from stages S8a to S11.** Ta, tapetum.

**Figure S2. Images showing ROS level differences between WT and *ms1-alb* anthers from stages S6 to S13 detected by** **H_2_DCFDA staining**

The corresponding developmental stages of each anther investigated are determined as evaluated in transverse sections.

**Figure S3. Images showing ROS level differences between WT and *p5126-ZmMs1* (#1) anthers from stages S6 to S13 detected by H_2_DCFDA staining**

The corresponding developmental stages of each anther investigated are determined as evaluated in transverse sections.

**Figure S4. Transcriptome analysis of *ms1-alb* and *p5126-ZmMs1* (#1) anthers from stages S5 to S12**

**(A)** PCA analysis of *ms1-alb* vs WT anther transcriptomes. **(B)** DEGs of *ms1-alb* vs WT anther transcriptomes. **(C)** PCA analysis of *p5126-ZmMs1* (#1) vs WT anther transcriptomes. **(D)** DEGs of *p5126-ZmMs1* (#1) vs WT anther transcriptomes.

**Figure S5.** **Four cysteine residues in the LOB domain of ZmMS1 are essential for its DNA binding ability and form disulfide bonds**

**(A)** Protein-DNA complex structure alignment. The ZmMS1-LOB homodimer was modeled by using Alphafold3, and the two ZmMS1-LOB monomers were colored in purple and cyan, respectively. The crystal structure of wheat TaRamosa2 LOB domain homodimer structure was retrieved from Protein Data Bank (https://www.rcsb.org/) under the accession ID number: 5LY0, and colored in red and orange. The B-form DNA motif recognized by ZmMS1 is colored in orange. **(B)** Purified MBP-ZmMS1 and MBP-ZmMS1-LOB proteins digested by the tobacco etch virus (TEV) protease to remove the MBP tag are shown on a SDS-PAGE gel. **(C)** EMSA analysis showing that the four cysteine residues in the LOB domain of ZmMS1 are indispensable for DNA binding. The selex probe contains the ZmMS1 binding motif CTCCGAAAATTTCGGAG. **(D)** Identification of inter- and intra-molecular disulfide bonds from the tryptic fragments of ZmMS1 based on mass spectrometry. Intra-molecular disulfide bonds are formed between C9 and C12, and C19 and C23. Inter-molecular disulfide bonds are formed among C9, C12, C19 and C23, and between C9 and C12. The purified ZmMS1 protein was pretreated with or without DTT as indicated.

**Figure S6. Lipid contents and expression of cutin- and sporopollenin-related genes in *ms1-alb*, *myb84*, *pksb* mutants and their corresponding WT anthers**

**(A**, **B)** Anther epidermal cutin and wax contents measured in *myb84* (A), *pksb* (B) and their WT anthers. **(C)** Comparison of cutin, wax, and internal lipid monomers per unit surface area in WT, *ms1*, *myb84* and *pksb* anthers at S13. The red and blue circles represent the increase and decrease of the cutin, wax, and internal lipid monomers, respectively. The size of the circles represents the magnitude of the increase or decrease. **(D)** Expression pattern changes of a cutin-related gene (*ZmLTL1*) in *ms1-alb*, *myb84*, *pksb* and their corresponding WT anthers from S8b–9 to S10 by RT-qPCR analysis. **(E)** Expression pattern changes of four sporopollenin-related genes in *ms1-alb*, *myb84*, *pksb* and their corresponding WT anthers by RT-qPCR analysis. **(F)** ChIP-seq peaks showing the enrichment of ZmMS1 in promoters of *ZmACOS-2* and *ZmPKSA-1*. Data in (**A**, **B**, **D**, **E**) represent mean ± *SD* (*n* = 3), and asterisks indicate significant differences (* *P*< 0.05, * *P*< 0.01, and ****P* < 0.001). A two-tailed Student’s *t*-test was used to determine *P* values.

**Figure S7. GO functional annotation of DEGs** **in *ms1-alb* and *p5126-ZmMs1* (#1)**

**(A)** GO functional annotation of up-regulated DEGs in *ms1-alb* vs WT anther transcriptomes from S8b–9 to S10. **(B)** GO functional annotation of down-regulated DEGs in *p5126-ZmMs1* (#1) vs WT anther transcriptomes from S7 to S9–10.

**Figure S8. GO functional annotation of up-regulated DEGs in *ms1-alb* and down-regulated in *p5126-ZmMs1* (#1) by anther transcriptome analysis**

**Figure S9. Phenotypic characterization of *pUbi-ZmMs1* (#1) transgenic maize plants**

**(A)** The *pUbi-ZmMs1* construct contains two functional modules: *pUbi-ZmMs1* and *pCaMV35S-Bar*. **(B**, **C)** Whole-plant morphology comparison of *pUbi-ZmMs1* (#1) transgenic line and WT (Hi II) grown in Sanya, Hainan province **(B)** and Beijing **(C)**, China. **(D**–**I)** Quantitative measurements of plant height **(D)**, ear height **(E)**, tassel length **(F)**, internode length **(G)**, number of internodes **(H)**, and leaf angle **(I)** of WT and five *pUbi-ZmMs1* transgenic maize lines at anthesis. Data in D–I represent mean ± *SD* (*n* = 10) and asterisks indicate significant differences (* *P*< 0.05, * *P*< 0.01, and ****P* < 0.001). A two-tailed Student’s *t*-test was used to determine *P* values.

**Figure S10.** **GA component contents and expression of GA- and ABA-related genes in *pUbi-ZmMs1* (#1) and WT stems**

**(A)** PCA analysis of the *pUbi-ZmMs1* (#1) vs WT transcriptomes. **(B)** GO functional annotation of down-regulated DEGs in *pUbi-ZmMs1* (#1) vs WT stem transcriptomes at jointing stage. **(C**, **D)** Upregulated **(C)** and downregulated **(D)** GA- and ABA-related genes in *pUbi-ZmMs1* (#1) by RT-qPCR analysis. **(E)** Content comparison of different GA components between WT and *pUbi-ZmMs1* (#1). Data in **(C**–**E)** represent mean ± *SD* (*n* = 3), and asterisks indicate significant differences (* *P*< 0.05, * *P*< 0.01, and ****P* < 0.001). A two-tailed Student’s *t*-test was used to determine *P* values. ns, not significant.

**Figure S11. SEM and transverse section observations of anther and pollen development in WT and *pUbi-ZmMs1***

**(A)** SEM analysis of anther and pollen in WT and *pUbi-ZmMs1* (#1) at stage S13. **(B)** Transverse section analysis of anthers in WT (Hi II) and *pUbi-ZmMs1* (#1) from S5 to S13. Dy, Dyad; E, epidermis; En, endothecium; ML, middle layer; MMC, microspore mother cell; Mp, mature pollen; Msp, microspore; Sp, sporogenous cell; T, tapetum; Tds, tetrads.

**Figure S12. Small pollen grains of *pUbi-ZmMs1* (#1) cannot transmit to the next generation**

**(A)** Pollen comparison of WT and *pUbi-ZmMs1* (#1) line based on I_2_-KI staining at stage S13. Green arrows indicate normal pollen grains, and red arrows indicate small pollen grains. **(B)** Segregation ratio of normal and dwarf plants in *pUbi-ZmMs1* (#1) (*pUbi-ZmMs1*/−) self-crossed progeny.

**Figure S13. Male-sterility stability analysis of homozygous *ms1-alb* mutation after introgression into various maize genetic backgrounds**

**(A)** Clustering graph of 547 maize inbred lines with various genetic backgrounds based on their genetic distances evaluated with 3072 SNP markers. **(B)** Distribution diagram of ratios of fertile to sterile individuals among the 547 F_2_ populations derived from 547 F_1_ crosses between *ms1-alb* mutant and each of the 547 maize lines as male parents. **(C)** Seven representative male-sterility tassel types of the 547 genetic backgrounds with genotype of *ms1-alb*/*ms1-alb* and their male-sterility pollen grains stained by I_2_-KI. **(D)** Seven representative F_2_ populations with ratios of fertile plants to sterile plants exhibiting skewed genetic ratios unlikely to fit the theoretical value of 3:1. **(E)** Genotyping individuals in the seven F_2_ populations in **(D)** by using one co-segregating marker with the *ms1-alb* mutation. Fertile (*ZmMs1*/*ZmMs1*), hybrid (*ZmMs1*/*ms1-alb*), and sterile (*ms1-alb*/*ms1-alb*) individuals are marked with yellow, blue, and red lines, respectively. This figure is a composite DNA gel image assembled from several separate gel runs, with dashed lines indicating the junctions where different gel images have been spliced together.

**Figure S14. Strategy for developing a MCS system using the *ms1-alb* mutant and *ZmMs1* gene and maize phenotypes of four representative *pMCS0107* transgenic events (0107-1, -3, -7, and -16)**

**(A)** Workflow of the MCS system based on the *ZmMs1* gene and *ms1-alb* mutant. **(B**–**E)** The pictures of maize ears were taken with a Canon EOS 700D digital camera (Canon, Japan) in the field under bright light **(B1**–**E1)**, green excitation light with red fluorescence filter I (GREEN.L, China) **(B2**–**E2)** and red fluorescence filter II (NIGHTSEA, USA) **(B3**-**E3)**, respectively.

**Figure S15. Ear display in field plot experiments of two representative hybrid combination pairs derived from WT and *ms1* mutant as female lines and Chang7/Ji853 (A**, **B) and Dan598 (C**, **D) as male lines**

These hybrids were planted in Zhucheng, Shandong province **(A**, **C)** and Puyang, Henan province **(B**, **D)** in China.

**Figure S16. The 27 hybrid combination pairs derived from crossing WT and *ms1* mutant with each of 27 different male lines were planted at the Zhucheng experimental station, Shandong province, China, for ear comparison**

**Figure S17. The 27 hybrid combination pairs derived from crossing WT and *ms1* mutant with each of 27 different male lines were planted at the Puyang experimental station, Henan province, China, for ear comparison**

**Figure S18. Investigation of two representative agronomic traits for heterosis assay of *ms1-alb* mutation**

**(A)** Analysis of plot yield of ten representative hybrid combination pairs from the crosses between the male sterile *ms1-alb* line or WT and ten maize male lines in Zhucheng, Shandong province. **(B**, **C)** Analysis of plant height of ten representative hybrid combination pairs from the crosses between male sterile *ms1-alb* line or WT and ten maize male lines in Zhucheng, Shandong province **(B)** and Puyang, Henan province **(C)** in China. Data represent means ± *SD* of 50 plants per cross with two replicates (**p* < 0.05; ***p* <0.01).

**Figure S19. Strategy for developing a *p5126-ZmMs1D*-based DGMS system**

**(A)** Tassels, anthers, pollen grains (stained by I_2_-KI), and ears of WT (B73) and four representative maize transgenic events (*p5126-ZmMs1D* #1 to #4) with dominant male sterility at T_1_ generation. **(B)** The pictures of a representative maize ear from *p5126-ZmMs1D* (#1) transgenic plants pollinated by B73 pollen were taken with a Canon EOS 700D digital camera (Canon, Japan) in the field under bright light, green excitation light with red fluorescence filter I (GREEN.L, China) and red fluorescence filter II (NIGHTSEA, USA), respectively. **(C)** Schematic diagram of the *p5126-ZmMs1D*-based DGMS system for maize breeding and hybrid seed production. MAS, marker-assisted selection.

**Figure S20. Dominant male-sterility stability assay of *p5126-ZmMs1D* (#1) transgenic line after introgression into various maize genetic backgrounds**

**(A)** Clustering graph of 379 maize lines with wide genetic backgrounds based on their genetic distances by scoring 3072 SNP markers. **(B)** Tassels, anthers, and pollen grains (stained by I_2_-KI) of *p5126-ZmMs1D* (#1) under four representative genetic backgrounds (A664, A956, A1308, and A1412) (*p5126-ZmMs1D* (#1)*/−*, S) and their corresponding male-fertile siblings (with genotype of *−/−*, F).

**Figure S21. Pollen grains (stained by I_2_-KI) of WT and *p5126-ZmMs1D* (#1) transgenic event under 95 genetic background lines**

All transgenic plants were completely male sterile and had no starch-filled pollen grains.

**Figure S22. Phenotype of WT and *p5126-ZmMs1D* (#1) event in ten maize genetic backgrounds at the BC_1_F_1_ generation**

**(A)** Tassels; **(B)** Pollen grains stained by 1% I_2_-KI; **(C**−**E)** Ears were photographed with a Canon EOS 700D digital camera (Canon, Japan) in the field in bright light **(C)**, and under green excitation light with red fluorescence filter I (GREEN.L, China) **(D)**, and red fluorescence filter II (NIGHTSEA, USA) (E).

**Figure S23. Phenotypic comparison of the corresponding recurrent parent lines and *p5126-ZmMs1D* (#1) transgenic event in different maize genetic backgrounds at the BC_2_F_1_ and BC_3_F_1_ generations**

**(A)** Pollen grains (stained by I_2_-KI) of WT (B73) and *p5126-ZmMs1D* (#1) transgenic event in ten maize genetic backgrounds at the BC_2_F_1_ generation. **(B)** The BC_3_F_1_ ears of *p5126-ZmMs1D* (#1) event backcrossed by five maize inbred lines were photographed with a Canon EOS 700D digital camera (Canon, Japan) in the field under bright light, green excitation light with red fluorescence filter I (GREEN.L, China) and red fluorescence filter II (NIGHTSEA, USA).

**Supplementary Tables**

**Table S1. Expression changes of 16 GA and ABA metabolism-related genes in *pUbi-ZmMs1* (#1) and WT stems based on RNA-seq analysis**

| **Gene name** | **Gene ID** | **WT-1** | **WT-2** | **WT-3** | ***pUBI-ZmMs1-1*** | ***pUBI-ZmMs1-2*** | ***pUBI-ZmMs1-3*** | **Log_2_FC** | **P value** |
| --- | --- | --- | --- | --- | --- | --- | --- | --- | --- |
| *ZmGA2ox8* | *Zm00001d038996* | 0.0263 | 0.1531 | 0.0970 | 1.9925 | 6.2943 | 2.9905 | 5.3202 | 0.0239 |
| *ZmGRAS37* | *Zm00001d005029* | 2.8934 | 4.7637 | 2.4033 | 7.4200 | 9.3706 | 12.2936 | 1.5310 | 0.0081 |
| *ZmGRAS46* | *Zm00001d044065* | 11.3243 | 17.6561 | 9.4354 | 23.4073 | 39.0760 | 44.6059 | 1.4789 | 0.0142 |
| *ZmGRAS50* | *Zm00001d041536* | 2.6444 | 2.6961 | 1.3043 | 6.0911 | 5.1697 | 7.3579 | 1.4856 | 0.0035 |
| *ZmGRAS58* | *Zm00001d029607* | 3.7177 | 3.4094 | 2.9681 | 5.7153 | 17.6742 | 10.5254 | 1.7476 | 0.0423 |
| *ZmSCRO1* | *Zm00001d052380* | 1.6315 | 3.3762 | 1.7350 | 9.4161 | 11.6894 | 6.9566 | 2.0563 | 0.0043 |
| *ZmZEP1* | *Zm00001d003513* | 0.3194 | 0.3805 | 0.3061 | 1.3786 | 2.3712 | 0.7741 | 2.1622 | 0.0328 |
| *ZmSnRK2.7* | *Zm00001d003659* | 3.8728 | 4.1202 | 8.4484 | 34.2533 | 20.9143 | 45.7163 | 2.6169 | 0.0092 |
| *ZmbZIP2* | *Zm00001d022613* | 0.8564 | 1.1375 | 2.0670 | 5.6381 | 19.1603 | 13.3507 | 3.2299 | 0.0223 |
| *ZmKAO* | *Zm00001d045563* | 9.8137 | 13.1942 | 2.2923 | 0.5969 | 3.5055 | 1.0103 | －2.3057 | 0.0574 |
| *ZmGA20ox4* | *Zm00001d013725* | 1.0850 | 1.5527 | 0.5426 | 0.6477 | 0.1089 | 0.2637 | －1.6344 | 0.0483 |
| *ZmGA13ox2* | *Zm00001d029031* | 4.6831 | 3.8230 | 3.2090 | 2.1432 | 0.9764 | 1.2663 | －1.4162 | 0.0058 |
| *ZmNPF2.9a* | *Zm00001d033528* | 13.9217 | 24.3584 | 25.2264 | 4.9630 | 6.0792 | 2.2247 | －2.2586 | 0.0059 |
| *ZmNPF2.9b* | *Zm00001d013529* | 87.2806 | 76.0149 | 52.4812 | 7.9766 | 6.0330 | 3.5852 | －3.6159 | 0.0015 |
| *ZmNPF3.1* | *Zm00001d045667* | 34.7504 | 54.7152 | 8.3643 | 10.4100 | 4.8385 | 8.2580 | －2.0569 | 0.0704 |
| *ZmPP2C16* | *Zm00001d005609* | 11.0817 | 7.7508 | 9.2211 | 1.5479 | 1.6216 | 0.2329 | －3.0413 | 0.0008 |

“FC” in log_2_FC represents fold change

**Table S2. Oligonucleotides used in this study**

| **Oligo name** | **Sequence (5'–3')** |
| --- | --- |
| ***p5126-ZmMs1* and *p5126-ZmMs1D* plasmids construction** | |
| p5126-F | ATCTTTCTGATTTCAACCAT |
| p5126-R | CGCAAAGCAACTTTGATTTG |
| Ms1-F | ATGACCGGCGGCGGCCGCGG |
| Ms1O-R | AGGGCATGCCGGATCCTCACCTGCAGGCGCTGCTCTTG |
| OcsMs1-F | CTGCAGGTGAGGATCCGGCATGCCCTGCTTTAATG |
| Ocster-R | CCGGCGGCCGCTAGCGTCTAGATCAATCAGTAAATTGAAC GGAGAATA |
| 5126ProF | ACATGATTACGAATTCATCTTTCTGATTTCAACCATTACC |
| Ms1Ocs-R | AGTTCTAGAGAAGCTTTCAATCAGTAAATTGAACGGAGAA |
| ***pUbi-ZmMs1* plasmid construction** | |
| Ms1-F1 | ATGACCGGCGGCGGCC |
| Ms1-R1 | TCACCTGCAGGCGCTGCTC |
| Ms1-F2 | GGGATGACCGGCGGCGGCC |
| Ms1-R2 | ATTGGATCCTCACCTGCAGGCGCTGCTC |
| **Quantitative PCR analysis** | |
| QUbi2-F | CGACAACGTGAAGGCGAAGA |
| QUbi2-R | ACGCAGATACCCAGGTACAGC |
| Q3KCAS-4-F | GCGTCTGGATGATCGGATTC |
| Q3KCAS-4-R | CTTGAGCACGTCGGGAATG |
| QAPV1-F | GCTCGGCTCCTTAGTTCGATG |
| QAPV1-R | ATTCATCGTTTGGTTTATGCGG |
| QLTL1-F | GCTGGTGGACATGATCAAGG |
| QLTL1-R | AAGTCCATGTGCATCCGGTA |
| QACOS5-2-F | CATCCCCAAGTCGGTGTCC |
| QACOS5-2-R | GTATAAGCAAGCACCCACCCAC |
| QPKSA-1-F | GCTGTTTGGTGATGGTGCAT |
| QPKSA-1-R | TTTGCCATCAATTACCTTATCAGTCC |
| QPKSA-2-F | CGGACCAGCAATACTAAACAGGC |
| QPKSA-2-R | ATTTCCGTAGTTCATCAAAGCCTTC |
| QMs25-F | TGATGGATCCTGTGGTCCTC |
| QMs25R | CGGGACCACATCAAGAACAC |
| QNPF2.9b-F | ATGGTTGGACAGGTTGGTGG |
| QNPF2.9b-R | CTCCTCTTTCATCAGCCACTTGA |
| QNPF3.1-F658 | AAGAGGAAGAAGAAGGCGGC |
| QNPF3.1-R656 | TCCTAGCACTGATGAGTACTTCCTTTC |
| QGA2OX8-F | CACCTGGCTCTCTATCTCGCT |
| QGA2OX8-R | GCCCACCGGATATTACTGCTAC |
| QGRAS37-F671 | GCCGTTGAGAGCTGAAATCG |
| QGRAS37-R66 | GGCACAAGAAACTCAAGCAGG |
| QGRAS46-F | CGGCAGTGAGGAGAGCAATA |
| QGRAS46-R | CAACGCCCTCTCAATCGAA |
| QGRAS50-F | CATCCTCTATGCCGTCTCAACA |
| QGRAS50-R | GTAACCCACTGAACACACTTGTCAC |
| QGRAS58-F | CTCGCGTGGAAGGAGCAG |
| QGRAS58-R | TGGAGCACGCATCATCTTGT |
| QSCRO1-F | TGCCGTTGATGAGACCTCTG |
| QSCRO1-R | CCAAACCGAGCAGCTACAGA |
| QZEP1-F | CGGAAGCGAACATGGTACCT |
| QZEP-R | GCACCTTCACCCGGAACATA |
| QSNRK2.7-F | ACGAGAAGCAGGTGAAGCAA |
| QSNRK2.7-R | CATCATCGGCTGGTACCACA |
| QBZIP2-F | GTGCCGATGGTGAGGAAGAC |
| QBIZP2-R | AACCACCTGCTTCGACTCATC |
| QKAO-F65 | CTAGGTGCCGGGTGAGGTA |
| QKAO-R669 | CGCTAGAGTTTCTGCTGGGCT |
| QGA20OX4-F666 | CAGCACCAGGAAGAACAGCA |
| QGA20OX4-R666 | GACATGGCGTGCTCTGAATCT |
| QGA13OX2-F65 | GTCTGTACTAAGAGGGGCACTGC |
| QGA13OX2-R674 | AAATTGTCCCCTCTCCGCAG |
| QNPF2.9a-F654 | TCTACTTCGTCATCTGCGCC |
| QNPF2.9a-R665 | AGCTGAGTCCCATTCATGCC |
| QPP2C16-F | CCGGAGGAGGGATCACTAGC |
| QPP2C16-R | GCGAGATCCTTTTGTGCACC |
| QMs1-F | CGTGTGGGGGCAATAATAATG |
| QMS1-R | CGTCGTCCGCTGTCATAGTAATAG |
| **Transient dual-luciferase assay** | |
| proZmMYB84:LUC-F | ATTACGCCAAGCTTGGTACCGGCCTCCGTGTTCTTTCTGA |
| proZmMYB84:LUC-R | TTGGCGTCTTCCATGGATCCCCTCGCCGGGCTGCTCTCGC |
| 35S-Ω:ZmMYB84-F | TGGCCACCATGGGCGAGCTCATGGGGCGGATCCCGTGCTG |
| 35S-Ω:ZmMYB84-R | GGGGATCCACTAGTTCTAGATCACACCATGTGATTGGTCA |
| 35S-Ω:ZmMs1-F | TGGCCACCATGGGCGAGCTCATGACCGGCGGCGGCCGC |
| 35S-Ω:ZmMs1-R | GGGGATCCACTAGTTCTAGATCACCTGCAGGCGCTGCT |
| APX12pro-F | ATTACGCCAAGCTTGGTACCGGTTAGCAATGAGCAGTGGTG |
| APX12pro-R | TTGGCGTCTTCCATGGATCCGGCACCGCGCGCGAGAG |
| DGATIII1pro-F | ATTACGCCAAGCTTGGTACCGCGCCAATGTTGAGGACTTG |
| DGATIII1pro-R | TTGGCGTCTTCCATGGATCCGGCCCGGAGCTTTCCCA |
| pcopro-F | ATTACGCCAAGCTTGGTACCTTGTTGCCGCGTGTCCC |
| pcopro-R | TTGGCGTCTTCCATGGATCCGGGTGTGAGCGAGTATGCG |
| TRXLpro-F | ATTACGCCAAGCTTGGTACCGGAGACGACGCTCGTCTTC |
| TRXLpro-R | TTGGCGTCTTCCATGGATCCGCAAACACCCTTTTTCGGC |
| NPF2.9bpro-F | ATTACGCCAAGCTTGGTACCCAACAACTGCAGCAACGAAC |
| NPF2.9bpro-R | TTGGCGTCTTCCATGGATCCTCCCGCGTCGGCCGGCT |
| NPF3.1bpro-F | ATTACGCCAAGCTTGGTACCGCCTTGTCGGTCCCTATCG |
| NPF3.1bpro-R | TTGGCGTCTTCCATGGATCCGGCTGCCGCCTTCTCC |
| **EMSA** | |
| ZmAPX12probe1F | CTGGGTGAGGCCGTGcGCGTGTGGCGCGGCGAGACTGGAGTCTAA |
| ZmAPX12probe1R | TTAGACTCCAGTCTCGCCGCGCCACACGCGCACGGCCTCACCCAG |
| Fam-ZmAPX12probe1F | CTGGGTGAGGCCGTGcGCGTGTGGCGCGGCGAGACTGGAGTCTAA |
| ZmTRXLprobeF | CGCGACCGTGCAGGTgCCGcGCGGGCAGGTGGATCTGATCGACTT |
| ZmTRXLprobeR | AAGTCGATCAGATCCACCTGCCCGCGCGGCACCTGCACGGTCGCG |
| Fam-ZmTRXLprobeF | CGCGACCGTGCAGGTgCCGcGCGGGCAGGTGGATCTGATCGACTT |
| Zmpcoprobe1F | CGAGCCGAAGGTGTGtCCGTCGCTTGAGGGGGTCCTCGGGCGAGA |
| Zmpcoprobe1R | TCTCGCCCGAGGACCCCCTCAAGCGACGGACACACCTTCGGCTCG |
| Fam-Zmpcoprobe1F | CGAGCCGAAGGTGTGtCCGTCGCTTGAGGGGGTCCTCGGGCGAGA |
| ZmDGATIII1-probeF | CGTGTGGCGTCGGCAgCCGGGCAGGTAGGAAAAGGTATCAGCGTC |
| ZmDGATIII1-probeR | GACGCTGATACCTTTTCCTACCTGCCCGGCTGCCGACGCCACACG |
| Fam-ZmDGATIII1-probeF | CGTGTGGCGTCGGCAgCCGGGCAGGTAGGAAAAGGTATCAGCGTC |
| ZmMYB84PROSbio2 | TACCACTCCGAGACAGCAGGCGCGTG |
| ZmMYB84PROS | TACCACTCCGAGACAGCAGGCGCGTG |
| ZmMYB84PROantiS2 | GCACGCGCCTGCTGTCTCGGAGTGGT |
| 84-ZmAPX12probeF | ATTAGTGGGTATGGAAAACCCGTTGATTACTCGAAA |
| 84-ZmAPX12probeR | TTTCGAGTAATCAACGGGTTTTCCATACCCACTAAT |
| 84-FAM-ZmAPX12probeF | ATTAGTGGGTATGGAAAACCCGTTGATTACTCGAAA |
| 84-ZmDGATIII1probeF | GCATATAAAGATGCCAACCCAAAGTAGCGTGAGTG |
| 84-ZmDGATIII1probeR | CACTCACGCTACTTTGGGTTGGCATCTTTATATGC |
| 84-FAM-ZmDGATIII1probeF | GCATATAAAGATGCCAACCCAAAGTAGCGTGAGTG |
| ZmNPF2.9bprobeF | CGCCTTGTTGCGTTGGCCGGACTCAAAGGCACCACCCTTCCTTGC |
| ZmNPF2.9bprobeR | GCAAGGAAGGGTGGTGCCTTTGAGTCCGGCCAACGCAACAAGGCG |
| FAM-ZmNPF2.9bprobeF | CGCCTTGTTGCGTTGGCCGGACTCAAAGGCACCACCCTTCCTTGC |
| ZmNPF3.1probeF | CCAACATACTGTGGGGGCGAAATTTCAGGCAGGTACTCAGTGATA |
| ZmNPF3.1probeR | TATCACTGAGTACCTGCCTGAAATTTCGCCCCCACAGTATGTTGG |
| FAM-ZmNPF3.1probeF | CCAACATACTGTGGGGGCGAAATTTCAGGCAGGTACTCAGTGATA |
| **LAC** | |
| yy01MS1lob-F72 | CGACGACAAGACCGTCACCATGACCGGCGGCGGC |
| yy01MS1lob-R699 | GAGGAGAAGAGCCGTCGGGCGCTGGACCAGAGCAG |
| **Recombinant protein expression** | |
| MBP-F | TAGCGCATTGGAAGTGGATAACGG |
| MBP-R | CGCATTGGATTGGAAGTACAGGT |
| ZmMs1MBP-F | TTCCAATCCAATGCGATGACCGGCGGCGGCCG |
| ZmMs1MBP-R | ACTTCCAATGCGCTATCACCTGCAGGCGCTGCTCTTG |
| ZmMs1lobMBP-R | ACTTCCAATGCGCTACTACTGCTGCTCGGCCAGCTGC |
| ZmMYB84MBP-F | TTCCAATCCAATGCGATGGGGCGGATCCCGTGCT |
| ZmMYB84MBP-R | TCCAATGCGCTATCACACCATGTGATTGGTCAGCTCGTCGGG |
| **Y2H** | |
| MS1LOB-AD.F | TGGCCATGGAGGCCAGTGAATTCCACATGACCGGCGGCGGCC |
| MS1LOB-BD.R | CTCGAGCTCGATGGATCCCGTATCAGGCGCTGGACCAGAGCAG |
| MS1GBK-F | ATATGGCCATGGAGGCCGAATTCCACATGACCGGCGGCGGCCG |
| BD-MS1LOB-R | GGTCGACGGATCCCTATCAGGCGCTGGACCAGAGCAG |

**Dataset S1. Differentially expressed genes for pairwise comparisons at each developmental stage of WT anthers (corresponding to *ms1-alb*) from developmental Stage 5 to Stage 12**

Please see Excel Dataset S1.

**Dataset S2. Differentially expressed genes for pairwise comparisons at each developmental stage of *ms1-alb* anthers from developmental Stage 5 to Stage 12**

Please see Excel Dataset S2.

**Dataset S3. Differentially expressed genes for pairwise comparisons at each developmental stage between WT and *ms1-alb* anthers from developmental Stage 5 to Stage 12**

Please see Excel Dataset S3.

**Dataset S4. Differentially expressed genes for pairwise comparisons at each developmental stage of WT anthers (corresponding to *p5126-ZmMs1*) from developmental Stage 5 to Stage 12**

Please see Excel Table S4.

**Dataset S5. Differentially expressed genes for pairwise comparisons at each developmental stage of *p5126-ZmMs1* anthers from developmental Stage 5 to Stage 12**

Please see Excel Dataset S5.

**Dataset S6. Differentially expressed genes for pairwise comparisons at each developmental stage between WT and *p5126-ZmMs1* anthers from developmental Stage 5 to Stage 12**

Please see Excel Dataset S6.

**Dataset S7. Expression levels of 11 ROS-scavenging genes in *ms1-alb*, *p5126-ZmMs1* (#1) and WT anthers based on RNA-seq analysis**

Please see Excel Dataset S7.

**Dataset S8. Differentially expressed genes in *pUbi-ZmMs1* (#1) identified by RNA sequencing**

Please see Excel Dataset S8.

**Dataset S9. Hormone content analysis in stems of *pUbi-ZmMs1* (#1) and WT**

Please see Excel Dataset S9.

**Dataset S10. The stability assay of *ms1-alb* male-sterility through segregation ratios of fertile plants to sterile plants in the 547 F_2_ populations**

Please see Excel Dataset S10.

**Dataset S11. Genotypes of the 547 maize inbred lines used for examining male-sterility stability of *ms1-alb* in the maize 3072 SNP chip**

Please see Excel Dataset S11.

**Dataset S12. The 17 agronomic trait investigation for testing the effects of *ms1-alb* mutation on maize field production**

Please see Excel Dataset S12.

**Dataset S13. Genotypes of the 379 maize inbred lines for examining male-sterility stability of *p5126-ZmMs1* (#1) in the maize 3072 SNP chip**

Please see Excel Dataset S13.
